# Chemical Constituents with Anti-Lipid Droplet Accumulation and Anti-Inflammatory Activity from *Elaeagnus glabra*

**DOI:** 10.3390/plants12162943

**Published:** 2023-08-14

**Authors:** Ju-Hsin Cheng, Ho-Cheng Wu, Chia-Hung Yen, Tsong-Long Hwang, Horng-Huey Ko, Hsun-Shuo Chang

**Affiliations:** 1 School of Pharmacy, College of Pharmacy, Kaohsiung Medical University, Kaohsiung 807, Taiwan; luluuuucyy0311@gmail.com (J.-H.C.); duncanwu762001@gmail.com (H.-C.W.); hhko@kmu.edu.tw (H.-H.K.); 2 Graduate Institute of Natural Products, College of Pharmacy, Kaohsiung Medical University, Kaohsiung 807, Taiwan; chyen@kmu.edu.tw; 3 Graduate Institute of Pharmacognosy, College of Pharmacy, Taipei Medical University, Taipei 110, Taiwan; 4 Drug Development and Value Creation Research Center, Kaohsiung Medical University, Kaohsiung 807, Taiwan; 5 Department of Medical Research, Kaohsiung Medical University Hospital, Kaohsiung 807, Taiwan; 6 Graduate Institute of Natural Products, College of Medicine, Chang Gung University, Taoyuan 333, Taiwan; htl@mail.cgu.edu.tw; 7 Research Center for Chinese Herbal Medicine, Graduate Institute of Health Industry Technology, College of Human Ecology, Chang Gung University of Science and Technology, Taoyuan 333, Taiwan; 8 Department of Anesthesiology, Chang Gung Memorial Hospital, Taoyuan 333, Taiwan

**Keywords:** *Elaeagnus glabra*, Elaeagnaceae, triterpenoid, flavonoid, anti-lipid droplet activity, anti-inflammation

## Abstract

Non-alcoholic fatty liver disease (NAFLD) is a type of steatosis caused by excess lipids accumulating in the liver. The prevalence of NAFLD has increased annually due to modern lifestyles and a lack of adequate medical treatment. Thus, we were motivated to investigate the bioactive components of Formosan plants that could attenuate lipid droplet (LD) accumulation. In a series of screenings of 3000 methanolic extracts from the Formosan plant extract bank for anti-LD accumulation activity, the methanolic extract of aerial parts of *Elaeagnus glabra* Thunb. showed excellent anti-LD accumulation activity. *E. glabra* is an evergreen shrub on which only a few phytochemical and biological studies have been conducted. Here, one new flavonoid (**1**), two new triterpenoids (**2** and **3**), and 35 known compounds (**4**–**38**) were isolated from the ethyl acetate layer of aerial parts of *E. glabra* via a bioassay-guided fractionation process. Their structures were characterized by 1D and 2D NMR, UV, IR, and MS data. Among the isolated compounds, methyl pheophorbide a (**37**) efficiently reduced the normalized LD content to 0.3% with a concentration of 20 μM in AML12 cell lines without significant cytotoxic effects. 3-*O*-(*E*)-Caffeoyloleanolic acid (**13**) and methyl pheophorbide a (**37**) showed inhibitory effects on superoxide anion generation or elastase release in fMLP/CB-treated human neutrophils (IC_50_ < 3.0 μM); they displayed effects similar to those of the positive control, namely, LY294002. These findings indicate that *E. glabra* can be used for developing a new botanical drug for managing LD accumulation and against inflammation-related diseases.

## 1. Introduction

Non-alcoholic fatty liver disease (NAFLD) is liver steatosis caused by a build-up of fat in the liver that is not caused by alcohol use. NAFLD is defined as a spectrum of liver diseases ranging from hepatic steatosis, intermediate lesions, and non-alcoholic steatohepatitis (NASH) to cirrhosis, depending on the definition and diagnosis result [1]. It is a hepatic event in the metabolic syndrome and is prevalent in industrialized and developing countries [2]. The prevalence of NAFLD has continuously risen annually in youths and adults [3] due to sedentary lifestyles and modern Western nutrition. The prevalence of NAFLD is approximately 25% in Europe [4], ~34% in the USA [5], and 12–51% in Taiwan [5]. Except for the hepatic complications, patients with NAFLD will not only be at a higher risk for developing other cardio-metabolic diseases (type 2 diabetes mellitus, cardiovascular disease, etc. [6]), chronic kidney diseases, and hepatocellular carcinoma but will also be at an increased risk of mortality due to these disorders [1]. Currently, no effective pharmacological treatments have been approved to treat NAFLD. The primary therapy for NAFLD is limited to weight loss and exercise [7]. Thus, novel pharmacological strategies against NAFLD are urgently needed.

Hepatic steatosis (fatty liver) is the first stage of NAFLD; this refers to an intracellular accumulation of lipids and subsequent formation of lipid droplets (LDs) in the cytoplasm of hepatocytes. Excess lipids in the liver are primarily neutral lipids; they are also triglycerides and cholesterol esters. In hepatocytes and other hepatic cells (for example, hepatic stellate cells and Kupffer cells), neutral lipids are stored in dynamic organelles called lipid droplets [8]. The stored LDs as buffers for fatty acids are then utilized in times of need to generate energy, membrane components, and signaling lipids [9]. The role of LDs as buffers for fatty acid availability may even extend to lipid exchange between cells in the same tissue. The excess fatty consumption breaks an imbalance between the formation and degradation of LDs and typically develops into severe physiological consequences, including lipodystrophy, NAFLD, obesity, cardiovascular disease, and type 2 diabetes mellitus [8,10]. From the current point of view, strategies for LD homeostasis seem to be the most promising for treating abnormal lipid accumulation and for the progression of liver diseases in patients with NAFLD [8].

**Figure 1:**
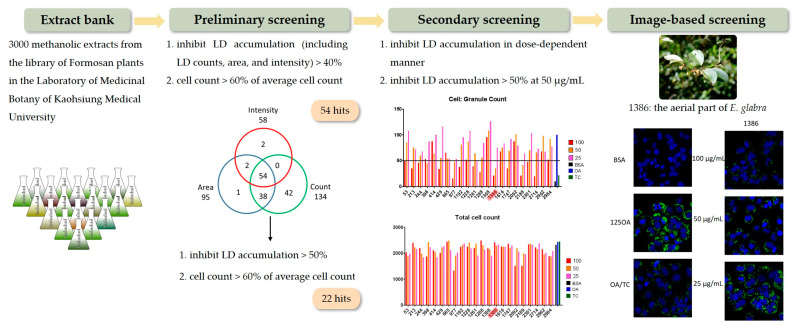
Use of the high-throughput screening platform for anti-LD candidate discovery from the Formosan methanolic extract bank and the results.

Natural products have been used as a medicinal agent source in previous centuries. Natural products and their derivatives have high chemical diversity, biochemical specificity, and other molecular properties that make them favorable as bioactivity screen resources. With the intention of identifying anti-LD agents, we screened over 3000 methanolic extracts from the library of Formosan plants in the Laboratory of Medicinal Botany of Kaohsiung Medical University [1]. Via high-throughput screening, extracts that inhibited LD accumulation (including LD counts, area, and intensity) by >40% without severe cytotoxicity (cell count >60% of average cell count in control wells) were considered hits in the primary screening. After that, we strengthened the inhibition criteria of LD accumulation to more than 50% and the cell count to >60% of the average cell count to obtain 22 hits. The extracts that showed a dose-dependent reduction in LD content at 50 μg/mL and a reduction of more than 50% in LD content were regarded as hits. Finally, the image-based platform was used to confirm anti-LD activity. After an evaluation, the methanolic extracts of aerial parts of *Elaeagnus glabra* Thunb. stood out for their potent inhibitory activity toward LD accumulation with little effect on cell viability (Figure 1).

*E. glabra* (Elaeagnaceae) is an evergreen shrub native to China, the Ryukyus in Japan, Korea, and Taiwan. Until now, only one antibacterial flavonoid, namely, (−)-epigallocatechin [11,12]; two steroids; and three triterpenes [13] have been identified from *E. glabra*. Based on the anti-LD accumulation screening results and the limited number of investigations of aerial parts from *E. glabra*, this study aimed to isolate components from *E. glabra* and evaluate their anti-LD accumulation effects.

## 2. Results and Discussion

In the current study, we focused on the chemical constituents from the methanolic extract of aerial parts of *E. glabra* and identified bioactive compounds with anti-LD accumulation activity. With the bioactivity-guided fractionation of the ethyl-acetate layer of aerial parts of *E. glabra*, three new compounds (**1**−**3**) and 35 known compounds (**4**−**38**) were successfully isolated (see Figure 2 and Supplementary Materials: Figure S1). The phytochemical spectra of compounds **1**–**3** are available in the Supplementary Materials (Figures S2–S28). In addition, some isolates were further examined for anti-LD accumulation effects in hepatic cell lines.

### 2.1. Structure Elucidation of Compounds ***1***−***3***

**Figure 3:**
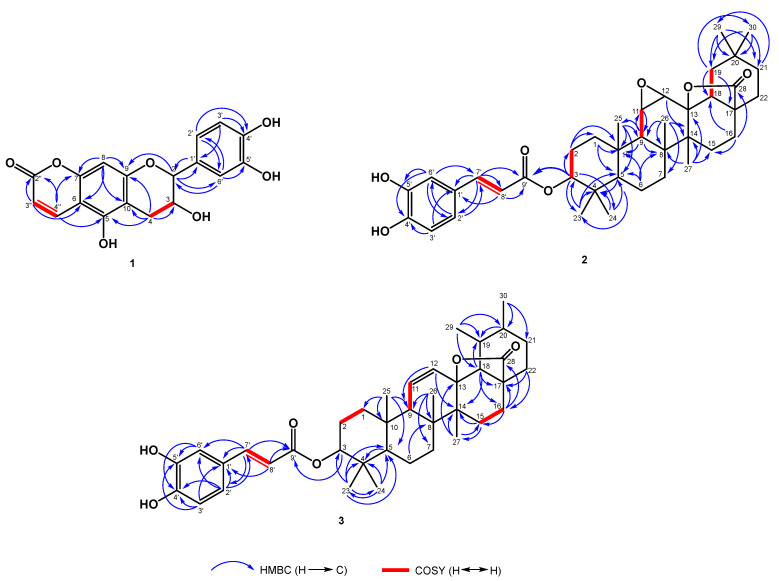
Key HMBC and COSY correlations of compounds **1**−**3**.

Compound **1** was isolated as a pale yellow amorphous solid with a negative optical rotation. Its molecular formula was determined to be C_18_H_14_O_7_ based on the results from HRESIMS, which is consistent with 12 hydrogen deficiencies. The hydroxy (3275 cm^−1^), carbonyl groups (1686 cm^−1^), and aromatic ring(s) (1606, 1519 cm^−1^) were observed in the IR spectrum. The UV absorption at 286 and 330 nm suggested the presence of a flavan moiety in **1** [14]. The characteristic flavan aromatic rings [*penta*-substituted aromatic ring: *δ*_H_ 6.35 (1H, s, H-8) and *δ*_C_ 153.4 (C-5), 103.4 (C-6), 156.2 (C-7), 95.7 (C-8), 163.1 (C-9), 105.6 (C-10); ABX system aromatic ring: *δ*_H_ 6.86 (1H, dd, *J* = 8.3, 2.3 Hz, H-6′), 6.80 (1H, d, *J* = 8.3 Hz, H-5′), 7.04 (1H, d, *J* = 2.3 Hz, H-2′) and *δ*_C_ 131.4 (C-1′), 115.2 (C-2′), 146.2 (C-3′), 146,1 (C-4′), 116.1 (C-5′), 119.3 (C-6′)] and a flavan 2*H*-pyran [ring C: *δ*_H_ 5.02 (1H, br s, H-2), 4.30 (1H, br dd, *J* = 4.2, 2.7 Hz, H-3), 2.87 (1H, dd, *J* = 17.0, 2.7 Hz, H-4b), 2.96 (1H, dd, *J* = 17.0, 4.2 Hz, H-4a) and *δ*_C_ 80.7 (C-2), 66.6 (C-3), 29.4 (C-4)] could also be found in the NMR spectra (Table 1). Further HMBC correlations between H-2/C-1′, C-2′ revealed that the ABX system aromatic ring occupied ring B, and the HMBC correlations between H-8/C-6, C-7, C-9, C-10, H-4/C-5, C-10, and H-2/C-9 indicated that the *penta*-substituted aromatic ring was located at ring A (Figure 3). Apart from the flavan moiety, the ^1^H- and ^13^C-NMR spectral data indicated the existence of a lactone ring [*δ*_H_ 6.07 (1H, d, *J* = 9.6 Hz, H-3″), 8.14 (1H, d, *J* = 9.6 Hz, H-4″); *δ*_C_ 103.4 (C-6), 156.2 (C-7), 164.5 (C-2″), 109.4 (C-3″), 141.2 (C-4″)]. Based on the HMBC correlation (Figure 3) between H-3″/C-6, H-4″/C-2″, C-5, and C-7, the lactone ring and flavan were connected with C-6 and C-7 to become the flavanocoumarin. For now, the above elucidations suggested the molecular formula of **1** as C_18_H_14_O_3_, showing losses of 64 atomic mass units as four oxygen atoms compared with its HRESIMS. Finally, based on the chemical shifts of C-3 (δ_C_ 66.6), C-5 (153.4), C-3′ (146.2), and C-4′ (146,1), four hydroxy groups were attached to C-3, C-5, C-3′, and C-4′, respectively. The small coupling constant of H-2/H-3 (*J*_2,3_ = 2.7 Hz) in **1** indicates that the relative configuration of C-2/C-3 was a 2,3-*cis*-configuration [15]. In accordance with the optical rotation rule [15,16], C-2/C-3 was in the (2*R*,3*R*)-form with a negative optical rotation. Accordingly, the structure of **1** was determined and named elaeagncoumarin.

**Figure 4:**
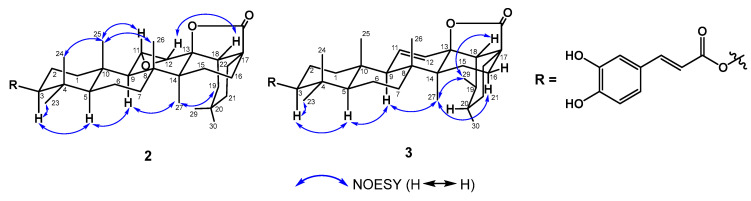
Key NOESY correlations of compounds **2** and **3**.

Compound **2**, a whitish powder, was established with its molecular formula of C_39_H_52_O_7_ using HRESIMS, accounting for 14 degrees of unsaturation. Compound **2** showed IR absorptions at 3385 (hydroxyl group(s)), 1768, and 1700 (carbonyl groups). The spectroscopic data of **2** (Table 2) are comparable with those of the literature compound 11α,12α-epoxy-3β-hydroxy-olean-13β,28-olide [17], except for the 3-OH group in 11α,12α-epoxy-3β-hydroxy-olean-13β,28-olide, which was changed to a *p*-*E*-coumaroyl moiety in **2**. Based on the ^1^H NMR data, the down-field shift in H-3 (*δ*_H_ 4.62) demonstrated that the *p*-*E*-coumaroyl moiety was located at C-3. A further HMBC correlation between H-3/C-9′ verified the junction between C-3 and the *p*-*E*-coumaroyl moiety (Figure 3). The NOESY plot of H-3/H-5, H-5/H-9, and H-9/H_3_-27 indicated that H-3, H-5, H-9, and H_3_-27 were in an α-axial configuration (Figure 4). H_2_-19 was assumed to be in an α-axial form due to the NOESY correlation between H_3_-27/H_2_-19, while H-18 was in the β-equatorial position in ring D. Therefore, H-13 was in the β-equatorial configuration based on the NOESY correlation between H-13/H_3_-18. In the NOESY spectra, the correlations between H_3_-24/H_3_-25, H_3_-25/H-11 and H_3_-26 suggested that H-11, H_3_-24, H_3_-25, and H_3_-26 occupied the β-axial configuration (Figure 4). The above NOESY correlations illustrated that compound **2** had the same relative configuration as the triterpene compound 11α,12α-epoxy-3β-hydroxy-olean-13β,28-olide [17]. Based on the above evidence, the structure of **2** was identified and named elaeagterpene A.

Compound **3** was obtained as a whitish powder. The analysis of the HRESIMS of **3** indicated the molecular formula C_39_H_52_O_6_, representing 14 degrees of unsaturation. IR absorptions at 3358, 1741, and 1704 cm^−1^ supported the presence of hydroxy and carbonyl groups. The physical data and NMR spectroscopic data of **3** and **2** (Table 2) implied their similar structure, except for differences in the substituents on C-11/C-12 (**2**: epoxide group; **3**: double bond), C-19 (**2**: H_2_; **3**: methyl group), and C-20 (**2**: dimethyl group; **3**: methyl group). The above signals indicated the ursane-type triterpenoid of **3**. The HMBC plots (Figure 3) of H_3_-25/C-1, C-5, C-9, and C-10; H_3_-26/C-7, C-8, and C-9; and H_3_-27/C-13, C-14, and C-15 could be used to assign the positions of C-8, C-9, C-13, and C-14. A further COSY correlation between H-9/H-11 and HMBC correlations between H-12/C-9 and C-14 verified that the double bond was located at C-11/C-12 (Figure 3). The doublet methyl groups [*δ*_H_ 1.04 (3H, d, *J* = 6.3 Hz, H-29), 0.96 (1H, *J* = 6.3 Hz, H-30)] were attached to C-19 and C-20 based on the HMBC correlations between H-29/C-18, C-19, C-20, H-30/C-20, and C-21. The relative configurations of **3** were indicated by the NOESY plots of H-3/H-5, H-5/H-9, and H-9/H_3_-27 (Figure 4) and a comparison with 3β,13-dihyroxyurs-11-en-28-oic acid γ-lactone [18]. Thus, the structure of compound **3** was assigned and named elaeagterpene B. 

Through a comparison of the experiments and reported spectroscopic data (${\left[\alpha \right]}_{D}$, UV, IR, NMR, and MS), 35 known compounds were identified as 11 triterpenoids: arjunolic acid (**4**) [19], alphitolic acid (**5**) [20], betulinic acid (**6**) [21], cleistocalyxic acid E (**7**), cleistocalyxic acid G (**8**) [22], lupeol (**9**) [23], pomolic acid (**10**) [24], ursolic acid (**11**) [25], 2α,3β,23-trihydroxy-11α,12α-epoxyolean-28,13β-olide (**12**) [19], 3-*O*-(*E*)-caffeoyloleanolic acid (**13**) [26], and (+)-3β-*O*-*trans*-caffeoyl betulinic acid (**14**) [27]; eight flavonoids: plumbocatechins B (**15**) [28,29], (−)-catechin (**16**) [30], dihydromyricetin (**17**) [31], (−)-epicatechin (**18**) [32], (−)-epigallocatechin (**19**) [33], (−)-gallocatechin (**20**) [34], naringenin (**21**) [35], and plumbocatechin A (**22**) [28]; five benzenoids: methyl galloate (**23**) [36], protocatechualdehyde (**24**) [37], syringaldehyde (**25**) [38], 4-(2-hydroxyethyl)benzoic acid (**26**) [39], and 4-hydroxy-3-methoxyphenyl)propane-1,2-diol (**27**) [40]; two *α*-tocopherol derivatives: α-tocopherol (**28**) [41] and 5-formyl-7,8-dimethyl tocol (**29**) [42]; three steroids: β-sitosterol (**30**) [43], β-sitosterone (**31**) [44], and ergosterol peroxide (**32**) [45]; two lignans: pinoresinol (**33**) [46] and syringaresinol (**34**) [47]; one alkanoid: 2,3-dihydroxypropyl hexadecanoate (**35**) [48]; one apocarotenoid: vomifoliol (**36**) [49]; one chlorophyll: methyl pheophorbide a (**37**) [50]; and one polyisoprenoid: ficarprenol-10 (**38**) [51].

### 2.2. Anti-LD Accumulation Activity of Compounds Isolated from Aerial Parts of E. glabra

**Figure 5:**
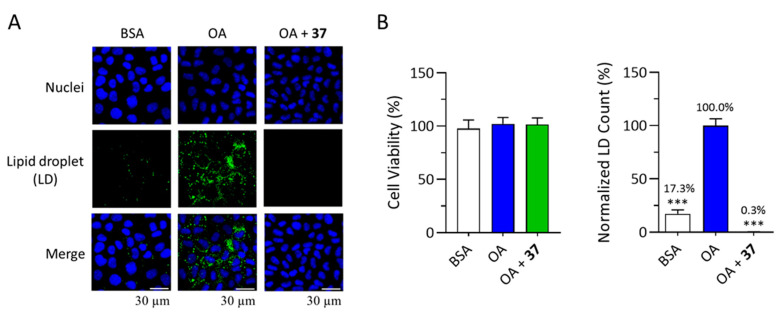
Effect of methyl pheophorbide a (**37**) on LD accumulation. (**A**) Representative images of the anti-LD formation activity of methyl pheophorbide a (**37**). (**B**) Quantification results of the LD assay and cell viability. AML12 cells were treated with BSA or OA (125 µM) with 20 µM methyl pheophorbide a (**37**) for 24 h. AML12 cells were used as a cell model for lipid accumulation—they were treated with 125 μM oleic acid (OA) for 24 h. Nuclei and LD were stained with Hoechst 33342 (blue) and BODIPY^®^ 493/503 (green), respectively. The asterisk indicates a significant difference from the solvent control cells (*** *p* < 0.001, one-way ANOVA).

In this study, twelve compounds present in sufficient amounts (**9**, **11**, **15**, **16**, **18**–**20**, **22**, **25**, **30**, **31**, and **37**) were evaluated for anti-LD accumulation activity (Table 3). To assess the anti-LD formation activity, the average LD counts/cell of bovine serum albumin (BSA)-conjugated oleic acid (OA) + drug vehicle (DMSO)-treated wells (hereinafter referred to as OA) were used as the standard for 100% fat loading, and triacsin C (TC) was used as the reference control. Compared with the vehicle control, methyl pheophorbide a (**37**) in 100 μg/mL did not affect the cell viability of the AML12 cell line, thus ensuring that the concentrations of methyl pheophorbide a (**37**) were safe. However, methyl pheophorbide a (**37**) significantly decreased the relative LD count, leaving only 0.3 ± 0.1%. Next, the representative images revealed the anti-LD accumulation activity directly. In Figure 5A, the blue pseudocolor indicates the nuclei area, and the green ones depict the LD area. Methyl pheophorbide a (**37**) at a concentration of 20 μM exhibited significant LD-accumulation-reducing effects (Figure 5A), with an LD content reduction of more than 90% without cytotoxicity (Figure 5B).

### 2.3. Anti-Inflammatory Activity of Compounds Isolated from Aerial Parts of E. glabra

**Figure 6:**
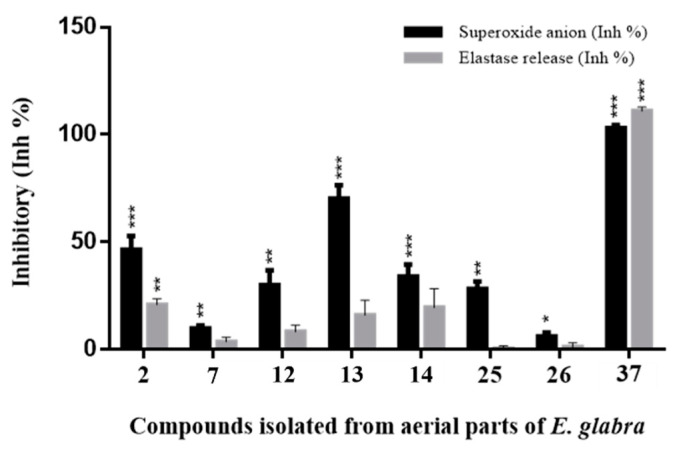
Preliminary screening of the inhibitory activities toward superoxide anion and elastase release of isolates from aerial parts of *E. glabra*. Percentage of inhibition (Inh%) at 10 μM. The results are presented as the mean ± S.E.M. (*n* = 3–5). * *p* < 0.05, ** *p* < 0.01, and *** *p* < 0.001 compared with the control (DMSO).

NAFLD is accompanied by inflammation. In the NASH stage, the liver is inflamed. As the NAFLD progresses, persistent inflammation causes scar tissue around the liver; this is called the fibrosis stage. After years of inflammation, liver damage occurs and becomes cirrhosis. To explore the potential of compounds for managing inflammation, eight compounds were preliminarily evaluated for their effects on neutrophil pro-inflammatory responses by suppressing fMLP/CB-induced superoxide anion generation and elastase release (Figure 6 and Table 4). Among them, compounds **13** and **37** showed significant inhibitory activity toward superoxide anion generation or elastase release (Figure 6). These two compounds were further evaluated for their anti-inflammatory activity IC_50_ values (Table 4). 3-*O*-(*E*)-Caffeoyloleanolic acid (**13**) showed superoxide anion inhibition with an IC_50_ value of 3.01 ± 0.58 μM, and methyl pheophorbide a (**37**) showed potent anti-inflammatory activity on both superoxide anion generation and elastase release with IC_50_ values of 1.29 ± 0.27 and 2.35 ± 0.12 μM, respectively.

There are approximately 40 *Elaeagnus* species in the world. Most isolates from *Elaeagnus* species are triterpenoids and flavonoids. The current study found 13 triterpenoids and 10 flavonoids in the aerial parts of *E. glabra*. Our chemical findings were consistent with the previous reports and can contribute to the chemotaxonomy of the *Elaeagnus* species. Furthermore, compounds **2** (oleanan-13β,28-olide-type) and **3** (urs-13β,28-olide-type) were triterpenes connected with caffeic acid, which has rarely been seen in *Elaeagnus*, even in natural sources. This finding not only sheds light on the structural diversity of *E. glabra* but also uncovers different types of compounds from the natural source. Furthermore, twenty compounds from aerial parts of *E. glabra* were found in *Elaeagnus* genus, including compounds **4**, **5**, **7**, **8**, **12**−**14**, **15**, **22**−**27**, **29**, **33**−**35**, **37**, and **38**, thus providing another natural source of the above compounds for further application. 

Our results from two different bioactivity models demonstrated that methyl pheophorbide a (**37**) can not only inhibit LD accumulation but also simultaneously display superoxide anion generation and elastase release inhibition. Methyl pheophorbide a (**37**) is a chlorophyll with several bioactivities in the literature, including antioxidant [52], cytotoxicity [52,53], phototoxicity [54], and iron-binding capacity [52]. To the best of our knowledge, this study demonstrated the first finding on the anti-LD accumulation activity of methyl pheophorbide a (**37**). It may be possible to further explore the anti-LD accumulation activity of chlorophylls accompanied by anti-inflammatory activity. 

## 3. Materials and Methods

### 3.1. General Experiment Procedures

Optical rotations were measured on a Jasco P-2000 polarimeter (Jasco, Kyoto, Japan), and IR spectra (ATR) were acquired with a Jasco FT/IR-4600 spectrometer. We recorded 1D (^1^H, ^13^C, DEPT) and 2D (COSY, NOESY, HSQC, HMBC) NMR spectra on a Varian Germini-2000 spectrometer (Varian, Inc. Vacuum Technologies, Lexington, MA, USA) operated at 200 (^1^H) and 50 MHz (^13^C), a Varian Unityplus-400 spectrometer (Varian, Inc. Vacuum Technologies, Lexington, MA, USA) operated at 400 (^1^H) and 100 MHz (^13^C), a Varian Mercuryplus-400 spectrometer (Varian, Inc. Vacuum Technologies, Lexington, MA, USA) operated at 400 (^1^H) and 100 MHz (^13^C), and a Varian VNMRS-600 spectrometer (Varian, Inc. Vacuum Technologies, Lexington, MA, USA) operated at 600 (^1^H) and 150 MHz (^13^C). Low-resolution mass spectra were obtained with POLARIS Q Thermo Finnigan (Thermo Fisher Scientific, Chicago, IL, USA), Waters ZQ 4000 (Waters, Milford, MA, USA), and VG Quattro GC/MS/MS/DS (Waters, Milford, MA, USA) mass spectrometers. EIMS was taken on a JEOL JMS-700 mass spectrometer (JEOL, Tokyo, Japan). High-resolution electrospray ionization mass spectroscopy (HRESIMS) was recorded on a Bruker APEX II mass spectrometer (Bruker, Karlsruhe, Germany) and VARIAN 901-MS (Varian, CA, USA). Silica gel (70–230 and 230–400 mesh; Silicycle, Quebec, QC, Canada) was used for column chromatography (CC), and silica gel 60 F254 (Merck, Darmstadt, Germany) and RP-18 F254S (Merck, Darmstadt, Germany) were used for thin layer chromatography (TLC) and preparative TLC, respectively, and visualized with a Ce_2_(SO_4_)_3_ aqueous solution. Further purification was performed using medium-performance liquid chromatography (MPLC; ceramic pump: VSP-3050; EYELA, Kyoto, Japan).

### 3.2. Plant Material

Aerial parts of *Elaeagnus glabra* Thunb. were collected in February 2018 in Sandimen Pingtung County, Taiwan, and identified by Prof. Ih-Sheng Chen. A voucher specimen (Chen 6318) was deposited with the herbarium of the College of Pharmacy, Kaohsiung Medical University, Kaohsiung, Taiwan.

### 3.3. Extraction and Isolation

Dried aerial parts (8.4 kg) of *Elaeagnus glabra* were extracted at room temperature with methanol (MeOH) (60 L) three times to yield a MeOH extract (610 g). The MeOH extract was suspended in water and partitioned with ethyl acetate (EtOAc) to yield a water layer (394 g) and an EtOAc layer (90 g). The EtOAc layer (90 g) was subjected to column chromatography (silica gel; *n*-hexane/acetone 95/5 to 33/67, then washed with 100% methanol) to yield ten fractions (Fr. 1–10). Fr. 2 (2.4 g) was subjected to column chromatography (silica gel, *n*-hexane/CH_2_Cl_2_ 4/1 to 3/2, column size: 2 × 30 cm) to yield seven fractions (Fr. 2-1~2-7). Fr. 2-1 was subjected to MPLC (silica gel, *n*-hexane/EtOAc 10/1, column size: 1.5 × 30 cm) to give eight fractions (Fr. 2-1-1~2-1-8). Fr. 2-1-2 was subjected to MPLC (RP-18; methanol/acetone 1:1; column size: 1.5 × 30 cm) to afford 11 fractions (Fr. 2-1-2-1~2-1-2-11) and compound **28** (34.8 mg). Fr. 2-1-2-11 was separated with prep. TLC (*n*-hexane/CH*_2_*Cl_2_/acetone 10/0.5/0.5) to afford compound **29** (0.5 mg). Fr. 2-6 was subjected to MPLC (silica gel, *n*-hexane/acetone 20/1, column size: 1 × 30 cm) to afford four fractions (Fr. 2-6-1~2-6-4). Fr. 2-6-2 was subjected to MPLC (RP-18, water/acetone 1/10, column size: 1 × 30 cm) to give five fractions (Fr. 2-6-2-1~2-6-2-5). Fr. 2-6-2-3 was separated with prep. TLC (*n*-hexane/acetone 8:1) to afford compound **38** (2.2 mg). Fr. 2-9 was subjected to MPLC (RP-18, water/methanol 1/10, column size: 1 × 30 cm) to afford nine fractions (Fr. 2-9-1~2-9-9) and compound **9** (73.7 mg). Fr. 2-11 was subjected to MPLC (RP-18, water/acetone 1/8, column size: 2 × 30 cm) to give five fractions (Fr. 2-11-1~2-11-5). Fr. 2-11-4 was subjected to MPLC (silica gel; *n*-hexane/acetone 20:1; column size: 1 × 30 cm) to afford compound **31** (34.8 mg). Fr. 3 was recrystallized from MeOH to give compound **30** (118.9 mg). Fr. 6 (1.7 g) was subjected to MPLC (RP-18; water/acetone 1/3; column size: 2 × 30 cm) to yield six fractions (Fr. 6-1~6-6). Fr. 6-2 was subjected to MPLC (silica gel, CH*_2_*Cl_2_/acetone 12/1, column size: 1.5 × 30 cm) to yield 15 fractions (Fr. 6-2-1~6-2-15). Fr. 6-2-10 was subjected to MPLC (silica gel, *n*-hexane/acetone 4/1, column size: 1 × 30 cm) to produce ten fractions (Fr. 6-2-10-1~6-2-10-10). Fr. 6-2-10-5 was subjected to MPLC (silica gel, *n*-hexane/EtOAc 3/1, column size: 1 × 30 cm) to afford eight fractions (Fr. 6-2-10-5-1~6-2-10-5-8). Fr. 6-2-10-5-4 was subjected to MPLC (silica gel, *n*-hexane/EtOAc 2:1, column size: 1 × 30 cm) to obtain six fractions (Fr. 6-2-10-5-4-1~6-2-10-5-4-6). Fr. 6-2-10-5-4-2 was further separated with prep. TLC (*n*-hexane/EtOAc 2/1) to give compound **10** (0.9 mg). Fr. 6-2-13 was subjected to MPLC (RP-18, water/acetonitrile 1/6, column size: 1 × 30 cm) to produce ten fractions (Fr. 6-2-13-1~6-2-13-10). Fr. 6-2-13-8 was separated with prep. TLC (CH*_2_*Cl_2_/Ethanol (EtOH) 12/1) to give compound **4** (2.1 mg). Fr. 6-3 was subjected to MPLC (silica gel, CH*_2_*Cl_2_/EtOAc 10/1, column size: 1.5 × 30 cm) to afford eight fractions (Fr. 6-3-1~6-3-8). Fr. 6-3-1 was separated with prep. TLC (*n*-hexane/acetone 4/1) to give compound **37** (7.1 mg). Fr. 6-3-2 was subjected to MPLC (silica gel, *n*-hexane/EtOAc 4/1, column size: 1 × 30 cm) to obtain seven fractions (Fr. 6-3-2-1~6-3-2-7). Fr. 6-3-2-1 was subjected to MPLC (silica gel, *n*-hexane/EtOAc 5/1, column size: 1 × 30 cm) to produce compound **6** (1.9 mg). Fr. 6-3-3 was subjected to MPLC (silica gel, *n*-hexane/CH*_2_*Cl_2_/acetone 7/1/1, column size: 1 × 30 cm) to obtain 11 fractions (Fr. 6-3-3-1~6-3-3-11). Fr. 6-3-3-4 was subjected to MPLC (RP-18, water/MeOH 1/6, column size: 1 × 30 cm) to afford compound **32** (3.2 mg). Fr. 6-3-4 was separated with Sephadex LH-20 (column size: 3 × 70 cm) and eluted with methanol to provide six fractions (6-3-4-1~6-3-4-6). Fr. 6-3-4-5 was subjected to MPLC (silica gel, *n*-hexane/acetone 6/1, column size: 1 × 30 cm) to obtain ten fractions (Fr. 6-3-4-5-1~6-3-4-5-10). Fr. 6-3-4-5-6 was subjected to MPLC (silica gel, *n*-hexane/EtOAc 2/1, column size: 1 × 30 cm) to give compound **35** (3.9 mg). Fr. 6-3-5 was subjected to MPLC (silica gel, *n*-hexane/EtOAc 4/1, column size: 1 × 30 cm) to produce compound **11** (1.7 mg). Fr. 8 (6.8 g) was subjected to column chromatography (silica gel, CH_2_Cl_2_/MeOH 35/1 to 20/1) to yield 11 fractions (Fr. 8-1~8-11). Fr. 8-4 was subjected to MPLC (silica gel, *n*-hexane/CH_2_Cl_2_/acetone 6/1/1, column size: 1.5 × 30 cm) to obtain 11 fractions (Fr. 8-4-1~8-4-11). Fr. 8-4-8 was subjected to prep. RP-18 TLC (water/acetone 1/4) to afford compound **3** (0.6 mg). Fr. 8-4-9 was subjected to prep. RP-18 TLC (water/acetone 1/4) to yield three fractions (Fr. 8-4-9-A~8-4-9-C). Fr. 8-4-9-B was subjected to MPLC (RP-18, water/acetonitrile 1/4, column size: 1 × 30 cm) to produce compound **2** (0.9 mg). Fr. 8-4-10 was subjected to MPLC (RP-18, water/acetone 2/1, column size: 1 × 30 cm) to afford compound **33** (1.0 mg). Fr. 8-4-11 was subjected to MPLC (RP-18, water/acetone 2/1, column size: 1 × 30 cm) to afford compound **34** (1.7 mg). Fr. 8-6 was subjected to MPLC (silica gel, *n*-hexane/EtOAc 2/1, column size: 1.5 × 30 cm) to yield six fractions (Fr. 8-6-1~8-6-6). Fr. 8-6-3 was subjected to MPLC (silica gel, *n*-hexane/CH_2_Cl_2_/EtOAc 2/1/1, column size: 1.5 × 30 cm) to afford compound **14** (9.6 mg). Fr. 8-6-3-3 was subjected to MPLC (silica gel, *n*-hexane/EtOAc 2/1, column size: 1 × 30 cm) to give compound **21** (0.6 mg) and compound **24** (0.7 mg). Fr. 8-6-4 was subjected to MPLC (RP-18, water/acetonitrile 1/5, column size: 1.5 × 30 cm) to produce compound **13** (6.6 mg). Fr. 8-6-6 was subjected to MPLC (RP-18, water/acetonitrile 2/1, column size: 1 × 30 cm) to obtain eleven fractions (Fr. 8-6-6-1~8-6-6-11). Fr. 8-6-6-2 was subjected to prep. RP-18 TLC (water/acetone 2/1) to yield three fractions (Fr. 8-6-6-2-A~8-6-6-2-C). Fr. 8-8 was subjected to MPLC (silica gel, CH_2_Cl_2_/EtOAc 2/3, column size: 2 × 30 cm) to obtain four fractions (Fr. 8-8-1~8-8-4). Fr. 8-8-3 was subjected to MPLC (RP-18, water/MeOH 1/3, column size: 1.5 × 30 cm) to yield nine fractions (Fr. 8-8-3-1~8-8-3-9). Fr. 8-8-3-2 was subjected to prep. TLC (CH_2_Cl_2_/EtOAc 1/1) to produce three fractions (Fr. 8-8-3-2-A~8-8-3-2-C) and compound **26** (2.5 mg). Fr. 8-8-3-2-C was further separated with MPLC (silica gel, CH_2_Cl_2_/EtOAc 1/3, column size: 1 × 30 cm) to give compound **36** (2.4 mg). Fr. 8-8-3-8 was subjected to prep. TLC (*n*-hexane/EtOAc 2/3) to produce compound **5** (6.5 mg). Fr. 9 (11.2 g) was subjected to column chromatography (silica gel, CH_2_Cl_2_/MeOH 12/1 to 6/1) to yield 13 fractions (Fr. 9-1~9-13). Fr. 9-5 was subjected to MPLC (silica gel, *n*-hexane/acetone 2/1, column size: 1.5 × 30 cm) to obtain 13 fractions (Fr. 9-5-1~9-5-13). Fr. 9-5-7 was subjected to MPLC (RP-18, water/MeOH 2/1, column size: 1 × 30 cm) to produce compound **23** (2.7 mg). Fr. 9-5-10 was subjected to MPLC (RP-18, water/MeOH 2/1, column size: 1 × 30 cm) to obtain 11 fractions (Fr. 9-5-10-1~9-5-10-11). Fr. 9-5-10-1 was subjected to MPLC (silica gel, *n*-hexane/CH_2_Cl_2_/MeOH 1/1/0.1, column size: 1 × 30 cm) to afford compound **27** (0.5 mg). Fr. 9-5-11 was subjected to MPLC (silica gel, *n*-hexane/CH_2_Cl_2_/MeOH 1/1/0.1, column size: 1.5 × 30 cm) to produce compound **12** (4.3 mg). Fr. 9-5-11-19 was subjected to column chromatography (silica gel, *n*-hexane/acetone 1.5/1) to yield nine fractions (Fr. 9-5-11-19-1~9-5-11-19-9). Fr. 9-5-11-19-7 was further separated with MPLC (RP-18, water/MeOH 1/1.5, column size: 1 × 30 cm) to give compound **7** (4.6 mg) and compound **8** (1.6 mg). Fr. 9-7 was subjected to MPLC (silica gel, *n*-hexane/acetone 1/1, column size: 1.5 × 30 cm) to obtain eight fractions (Fr. 9-7-1~9-7-8). Fr. 9-7-6 was subjected to MPLC (RP-18, water/MeOH 1.5/1, column size: 1 × 30 cm) to yield 21 fractions (Fr. 9-7-6-1~9-7-6-21). Fr. 9-7-6-18 was subjected to MPLC (silica gel, CH_2_Cl_2_/acetone 2/1, column size: 1 × 30 cm) to afford compound **1** (0.9 mg). Fr. 9-9 was subjected to MPLC (silica gel, CH_2_Cl_2_/EtOAc 1/1, column size: 1.5 × 30 cm) to obtain ten fractions (Fr. 9-9-1~9-9-10). Fr. 9-9-3 was subjected to MPLC (RP-18, water/acetonitrile 3/1, column size: 1 × 30 cm) to yield eight fractions (Fr. 9-9-3-1~9-9-3-8). Fr. 9-9-3-3 was further separated with prep. RP-18 TLC (water/MeOH 1.5/1) to give compound **15** (3.6 mg) and compound **17** (1.3 mg). Fr. 9-9-4 was subjected to prep. RP-18 TLC (water/MeOH 1.5/1) to afford compound **25** (1.5 mg). Fr. 9-10 was subjected to MPLC (silica gel, CH_2_Cl_2_/MeOH 7/1, column size: 2.5 × 30 cm) to obtain nine fractions (Fr. 9-10-1~9-10-9). Fr. 9-10-2 was subjected to MPLC (RP-18, water/MeOH 2/1) to yield two fractions (Fr. 9-10-2-1~9-10-2-2). Fr. 9-10-2-1 was further separated with prep. RP-18 TLC (water/MeOH 1/1) to yield six fractions (Fr. 9-10-2-1-A~9-10-2-1-F). Fr. 9-10-2-1-D was subjected to MPLC (silica gel, CH_2_Cl_2_/EtOAc 1/1, column size: 1 × 30 cm) to afford compound **22** (4.6 mg). Fr. 9-10-3 was subjected to prep. RP-18 TLC (water/MeOH 2/1) to afford compound **18** (4.1 mg). Fr. 9-10-5 was subjected to MPLC (silica gel, CH_2_Cl_2_/MeOH 6/1) to yield ten fractions (Fr. 9-10-5-1~9-10-5-10). Fr. 9-10-5-5 was further separated with prep. RP-18 TLC (water/acetonitrile 3/1) to afford compound **16** (1.6 mg). Fr. 9-10-5-7 was subjected to prep. RP-18 TLC (water/acetonitrile 3/1) to afford compound **19** (12.9 mg) and compound **20** (1.2 mg).

### 3.4. Spectroscopic Data of New Compounds

#### 3.4.1. Elaeagncoumarin (**1**)

##### [(7*R*,8*R*)-8-(3,4-Dihydroxyphenyl)-5,7-dihydroxy-7,8-dihydro-2*H*,6*H*-pyrano[3,2-g]chromen-2-one]

Pale yellow amorphous solid; ${\left[\alpha \right]}_{D}^{24}$ − 24.8 (*c* 0.04, MeOH); UV *λ*_max_ (MeOH) (log *ε*): 286 (3.07), 330 (4.00) nm; UV *λ*_max_ (MeOH + KOH) (log *ε*): 383 (4.05) nm; IR *v*_max_ (ATR): 3275 (OH), 1686 (carbonyl group), 1606, 1519 (aromatic ring) cm^−1^; ESIMS *m*/*z* 365 [M + Na]^+^; HRESIMS *m*/*z* 365.06340 [M + Na]^+^ (calcd. for C_18_H_14_NaO_7_, 365.06317); ^1^H-NMR and ^13^C-NMR (Table 1).

#### 3.4.2. Elaeagterpene A (**2**)

##### [(3*S*,4a*R*,6a*R*,6b*S*,8a*S*,12a*R*,12b*S*,12c*S*,13a*S*,13b*R*,13cS)-4,4,6a,6b,11,11,13c-Heptamethyl-15-oxooctadecahydro-1*H*,9*H*-12b,8a-(epoxymethano)piceno [13,14-b]oxiren-3-yl(*E*)-3-(3,4-dihydroxyphenyl)acrylate]

Whitish powder; ${\left[\alpha \right]}_{D}^{23}$ + 21.8 (*c* 0.25, MeOH); UV *λ*_max_ (MeOH) (log *ε*): 200 (3.99), 238 (3.70), 290 (3.74), 328 (3.88) nm; UV *λ*_max_ (MeOH + KOH) (log *ε*): 202 (4.82), 257 (4.67), 398 (3.81) nm; IR *v*_max_ (ATR): 3385 (OH), 1768, 1700 (carbonyl group) cm^−1^; ESIMS *m*/*z* 655 [M + Na]^+^; HRESIMS *m*/*z* 655.36022 [M + Na]^+^ (calcd. for C_39_H_52_NaO_7_, 655.36053); ^1^H-NMR and ^13^C-NMR (Table 2).

#### 3.4.3. Elaeagterpene B (**3**)

##### [(3*S*,4a*R*,6a*R*,6b*S*,8a*S*,11*R*,12*S*,12a*R*,12b*S*,14a*R*,14b*S*)-4,4,6a,6b,11,12,14b-Heptamethyl-16-oxo-2,3,4,4a,5,6,6a,6b,7,8,10,11,12,12a,14a,14b-hexadecahydro-1*H*,9*H*-12b,8a-(epoxymethano)picen-3-yl(*E*)-3-(3,4-dihydroxyphenyl)acrylate]

Whitish powder; ${\left[\alpha \right]}_{D}^{23}$ + 42.3 (*c* 0.09, MeOH); UV *λ*_max_ (MeOH) (log *ε*): 202 (4.47), 250 (3.51), 298 (4.24), 326 (4.34) nm; UV *λ*_max_ (MeOH + KOH) (log *ε*): 206 (5.05), 257 (4.08), 310 (3.96), 372 (4.46) nm; IR *v*_max_ (ATR): 3358 (OH), 1741, 1704 (carbonyl group) cm^−1^; ESIMS *m*/*z* 639 [M + Na]^+^; HRESIMS *m*/*z* 639.36548 [M + Na]^+^ (calcd. for C_39_H_52_NaO_6_, 639.36561); ^1^H-NMR and ^13^C-NMR (Table 2).

### 3.5. Cell Line

Cells were cultured as described previously [55]. The medium for AML12 (BCRC 60326; Bioresources Collection and Research Center (BCRC), Hsinchu, Taiwan) was a 1:1 mixture of DMEM and Ham’s F12 medium with 10% FBS and 1× ITS-A supplement (Thermo Fisher Scientific).

### 3.6. LD Assay

The LD accumulation of the test compounds was evaluated according to the studies published by co-author Professor Chia-Hung Yen [1]. The LD accumulation was detected by BODIPY^®^ 493/503 dye (Thermo Fisher Scientific). LD accumulation was achieved by treating cells with OA conjugated to BSA. All data were analyzed with GraphPad Prism 6.01 software (La Jolla, CA, USA). One-way analysis of variance (ANOVA) followed by Tukey’s comparison test was used to compare differences between multiple groups. A *p*-value < 0.05 was considered statistically significant.

### 3.7. Superoxide Anion and Elastase Release Assays

Neutrophils were collected from healthy adults aged 20–35 and isolated using Ficoll-Paque density separation. The study was approved by Chang Gung Memorial Hospital’s Institutional Review Board (IRB No. 201902217A3) following the Declaration of Helsinki guidelines. The methods for testing the effects of isolates on superoxide anion generation and elastase release in neutrophils were based on studies published by Professor Tsong-Long Hwang [56,57]. Ferricytochrome c (0.6 mg/mL) was used to measure superoxide anion. Elastase substrate (methoxysuccinyl-Ala-Ala-Pro-Val-p-nitroanilide, 100 µM; Merck) was employed to detect the elastase release. The elastase level was detected using a spectrophotometer at OD 405 nm. A PI3K inhibitor, namely, LY29002, was used as a positive control in the neutrophil assays. All assays were repeated at least three times. Results are presented as the mean ± standard error of the mean (S.E.M.). Student′s *t*-test was used to compare the test isolates with a DMSO (0.1%) control. A probability of less than 0.05 was considered significant.

## 4. Conclusions

This study used 3000 Formosan plant extracts as a natural product library for a high throughput screening for anti-NAFLD drug discoveries. The aerial parts of *E. glabra* were selected as the research material due to their significant anti-LD accumulation activity without severe cytotoxicity. In the current study, we uncovered three new compounds and 35 known compounds from aerial parts of *E. glabra*. The skeletons of triterpenoids and flavonoids were a major part of this study, which was consistent with the chemotaxonomy of Elaeagnaceae. The bioactivity results indicated that chlorophyll (compound **37**) could reduce LD accumulation. This is the first report on the anti-LD accumulation activity of aerial parts of *E. glabra*. Furthermore, 3-*O*-(*E*)-caffeoyloleanolic acid (**13**) and methyl pheophorbide a (**37**) showed potent inhibitory activities on superoxide anion or elastase release in human neutrophils, displaying effects similar to those of the positive control, namely, LY294002. Our findings from the current study support the idea that methyl pheophorbide a (**37**) can both reduce LD accumulation and show anti-inflammatory activity, thus helping to manage NAFLD progression and related liver inflammation. Taken together, this study revealed the chemical characteristics and bioactivities of *E. glabra*, providing substantive evidence that *E. glabra* could be used for the development of anti-NAFLD drugs.

## Figures and Tables

**Figure 2:**
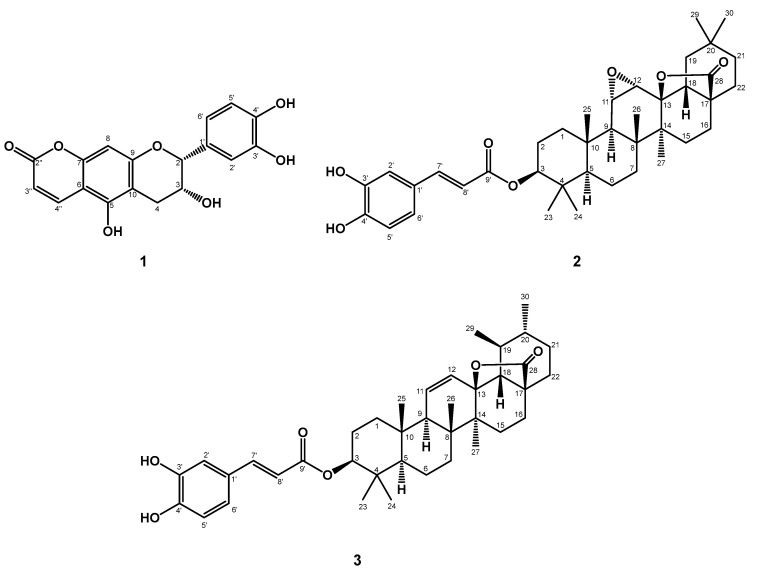
Structures of compounds **1**−**3**.

**Table 1:** ^1^H NMR (600 MHz, CD_3_OD) and ^13^C NMR (150 MHz, CD_3_OD) data of compound **1**.

Position	1	
	*δ*_H_ (*J* in Hz)	*δ*_C_
1	–	–
2	5.02, br s	80.7
3	4.30, br dd (4.2, 2.7)	66.6
4a	2.96, dd (17.0, 4.2)	29.4
4b	2.87, dd (17.0, 2.7)	
5	–	153.4
6	–	103.4
7	–	156.2
8	6.35, s	95.7
9	–	163.1
10	–	105.6
1′	–	131.4
2′	7.04, d (2.3)	115.2
3′	–	146.2
4′	–	146.1
5′	6.80, d (8.3)	116.1
6′	6.86, dd (8.3, 2.3)	119.3
2″	–	164.5
3″	6.07, d (9.6)	109.4
4″	8.14, d (9.6)	141.2

**Table 2:** ^1^H NMR (600 MHz, CD_3_OD) and ^13^C NMR (150 MHz, CD_3_OD) data of compounds **2** and **3**.

Position	2		3	
	*δ*_H_ (*J* in Hz)	*δ*_C_	*δ*_H_ (*J* in Hz)	*δ*_C_
1	1.60, m	38.9	1.12, m/1.93, m	39.0
2	1.60, m/1.81, m	24.4	1.50, m/1.76, m	32.5
3	4.62, dd (11.4, 4.8)	82.0	4.60, dd (11.4, 4.8)	82.0
4	–	39.1	–	39.3
5	0.94, m	56.0	0.99, m	56.0
6	1.60, m	18.6	1.67, m	18.7
7	1.20, m/1.41, m	32.2	1.30, m	32.3
8	–	42.6	–	43.2
9	1.66, br s	52.0	2.11, br s	54.3
10	–	37.7	–	37.5
11	3.12, dd (3.9, 1.8)	53.8	5.60, dd (10.5, 3.3)	130.0
12	3.06, d (3.9)	58.2	6.06, dd (10.5, 1.5)	134.8
13	–	89.4	–	91.9
14	–	41.8	–	43.0
15	1.15, m	27.8	1.67, m	26.6
16	1.29, m/2.28, m	22.3	1.30, m/2.30, ddd (18.9, 13.5, 5.7)	23.9
17	–	45.4	–	46.6
18	2.40, dd (13.8, 3.0)	50.8	1.70, d (11.4)	61.8
19	1.29, m/1.99, m	38.9	1.12, m	39.2
20	–	32.3	0.95, m	41.5
21	1.29, m/1.47, ddd (18.6, 14.0, 4.4)	35.2	1.35, m/1.59, m	31.8
22	1.60, m/1.70, dd (14.0, 4.4)	28.2	1.76, m/1.81, m	24.5
23	0.92, s	16.9	0.92, s	16.7
24	0.99, s	28.3	0.98, s	28.3
25	1.14, s	17.7	1.01, s	15.8
26	1.08, s	20.7	1.06, s	19.6
27	1.17, s	19.3	1.25, s	16.6
28	–	181.6	–	182.6
29	0.96, s	24.0	1.04, d (6.3)	18.3
30	1.02, s	33.5	0.96, d (6.3)	19.4
1′	–	127.7	–	127.7
2′	7.04, d (2.3)	115.1	7.04, d (2.4)	115.1
3′	–	146.85	–	146.85
4′	–	149.6	–	149.6
5′	6.78, d (8.6)	116.5	6.78, d (8.4)	116.5
6′	6.95, dd (8.6, 2.3)	122.9	6.95, dd (8.4, 2.4)	122.9
7′	7.54, d (15.9)	146.78	7.53, d (15.9)	146.75
8′	6.26, d (15.9)	115.5	6.25, d (15.9)	115.5
9′	–	169.1	–	169.2

**Table 3:** Anti-lipid droplet accumulation activity of compounds isolated from *E. glabra*.

Compound (100 μg/mL)	Relative Lipid Droplet Count (%) ^a^	Cell Viability (%, 24h, DAPI Image) ^b^
lupeol (**9**)	95.2 ± 1.8	113.7 ± 3.6
ursolic acid (**11**)	99.2 ± 4	146.5 ± 4.5
plumbocatechins B (**15**)	105.9 ± 7.8	132.5 ± 11.8
(−)-catechin (**16**)	110.7 ± 5.1	154 ± 12.3
(−)-epicatechin (**18**)	115.9 ± 6	145.9 ± 10.3
(−)-epigallocatechin (**19**)	93.5 ± 6.6	127.2 ± 22.6
(−)-gallocatechin (**20**)	116.7 ± 7.8	153.9 ± 6.2
plumbocatechin A (**22**)	118.2 ± 3.1	147.8 ± 9.6
syringaldehyde (**25**)	102 ± 5	137.8 ± 5.7
β-sitosterol (**30**)	112.7 ± 7.6	128.5 ± 9.1
β-sitosterone (**31**)	121.5 ± 4.3	135.3 ± 7.3
methyl pheophorbide a (**37**)	0.3 ± 0.1	99.5 ± 5.4
TC ^c^	60.6 ± 0.2	126.1 ± 6.1

^a^ Relative LD counts—the average LD counts/cells of oleic-acid-treated groups were used as 100% fat-loading in the AML12 cell line. ^b^ Cell viability—the average nucleus counts of DMSO were used as 100% cell viability in the AML12 cell line. ^c^ Triacsin C (TC) is an inhibitor of long fatty acyl CoA synthetase and was used as a positive control for LD inhibition. The drug concentration was 8 nM.

**Table 4:** Inhibitory effects of the active compounds on superoxide anion generation and elastase release in fMLP/CB-induced human neutrophils.

Compound	Superoxide Anion IC_50_ (μM)	Elastase Release IC_50_ (μM)
elaeagterpene A (**2**)	>10	>10
cleistocalyxic acid E (**7**)	>10	>10
2α,3β,23-trihydroxy-11α,12α-epoxyolean-28,13β-olide (**12**)	>10	>10
3-*O*-(*E*)-caffeoyloleanolic acid (**13**)	3.01 ± 0.58	>10
(+)-3β-*O*-*trans*-caffeoyl betulinic acid (**14**)	>10	>10
syringaldehyde (**25**)	>10	>10
4-(2-hydroxyethyl)benzoic acid (**26**)	>10	>10
methyl pheophorbide a (**37**)	1.29 ± 0.27	2.35 ± 0.12
^#^LY294002	1.29 ± 0.52	2.61 ± 0.72

The results are presented as the mean ± S.E.M. (*n* = 3–4). The compound was not considered anti-inflammatory when the IC_50_ value was >10 µM. ^#^ A phosphatidylinositol-3-kinase inhibitor (LY294002) was used as a positive control.

## Data Availability

The data presented in this study are available in the Supplementary Materials.

## Supplementary Materials

The following supporting information can be downloaded from https://www.mdpi.com/article/10.3390/plants12162943/s1, Figure S1: Structures of known compounds **4**–**38**; Figures S2–S28: The phytochemical spectra of compounds **1**–**3**.
