# Supplementary material for: Chemical Constituents with Anti-Lipid Droplet Accumulation and Anti-Inflammatory Activity from Elaeagnus glabra
Source: Plants (Basel). 2023 Aug 14;12(16):2943. doi: 10.3390/plants12162943 (PMC10458971; doi:10.3390/plants12162943)
Supplement: Supplementary file 1 [file plants-12-02943-s001.zip › plants-2542497-supplementary.pdf]

Supplementary Materials:

Chemical Constituents with Anti-Lipid Droplet Accumulation and Anti-Inflammatory Activity from *Elaeagnus glabra*

Ju-Hsin Cheng [1,2], Ho-Cheng Wu [1,3], Chia-Hung Yen [2,4,5], Tsong-Long Hwang [6,7,8], Horng-Huey Ko [1,4,5] and Hsun-Shuo Chang [1,2,4,5,*]

1 School of Pharmacy, College of Pharmacy, KaohsiungMedical University, Kaohsiung 807, Taiwan; luluuuucyy0311@gmail.com (J.-H.C.); duncanwu762001@gmail.com (H.-C.W.); hhko@kmu.edu.tw (H.-H.K.)
2 Graduate Institute of Natural Products, College of Pharmacy, Kaohsiung Medical University, Kaohsiung 807, Taiwan; chyen@kmu.edu.tw
3 Graduate Institute of Pharmacognosy, College of Pharmacy, Taipei Medical University, Taipei 110, Taiwan
4 Drug Development and Value Creation Research Center, Kaohsiung Medical University, Kaohsiung 807, Taiwan
5 Department of Medical Research, Kaohsiung Medical University Hospital, Kaohsiung 807, Taiwan
6 Graduate Institute of Natural Products, College of Medicine, Chang Gung University, Taoyuan 333, Taiwan; htl@mail.cgu.edu.tw
7 Research Center for Chinese Herbal Medicine, Graduate Institute of Health Industry Technology, College of Human Ecology, Chang Gung University of Science and Technology, Taoyuan 333, Taiwan
8 Department of Anesthesiology, Chang Gung Memorial Hospital, Taoyuan 333, Taiwan
* Correspondence: hschang@kmu.edu.tw; Tel.: +886-7-312-1101 (ext. 2664).

The List of Supplementary Materials

Figure S1. Structures of known compounds 4–38. 3

Figure S2. ^{1}H NMR (600 MHz, CD_3OD) spectrum of 1 4

Figure S3. ^{13}C NMR (150 MHz, CD_3OD) spectrum of 1 4

Figure S4. DEPT spectrum of 1 5

Figure S5. COSY spectrum of 1 5

Figure S6. NOESY spectrum of 1 6

Figure S7. HSQC spectrum of 1 6

Figure S8. HMBC spectrum of 1 7

Figure S9. ESIMS spectrum of 1 7

Figure S10. HRESIMS spectrum of 1 8

Figure S11. ^{1}H NMR (600 MHz, CD_3OD) spectrum of 2 8

Figure S12. ^{13}C NMR (150 MHz, CD_3OD) spectrum of 2 9

Figure S13. DEPT spectrum of 2 9

Figure S14. COSY spectrum of 2 10

Figure S15. NOESY spectrum of 2 10

Figure S16. HSQC spectrum of 2 11

Figure S17. HMBC spectrum of 2 11

Figure S18. ESI spectrum of 2 12

Figure S19. HRESI spectrum of 2 12

Figure S20. ^{1}H NMR (600 MHz, CD_3OD) spectrum of 3 13

Figure S21. ^{13}C NMR (150 MHz, CD_3OD) spectrum of 3 13

Figure S22. DEPT spectrum of 3 14

Figure S23. COSY spectrum of 3 14

Figure S24. NOESY spectrum of 3 15

Figure S25. HSQC spectrum of 3 15

Figure S26. HMBC spectrum of 3 .... **16**

Figure S27. ESIMS spectrum of 3 .... **16**

Figure S28. HRESIMS spectrum of 3 .... **17**

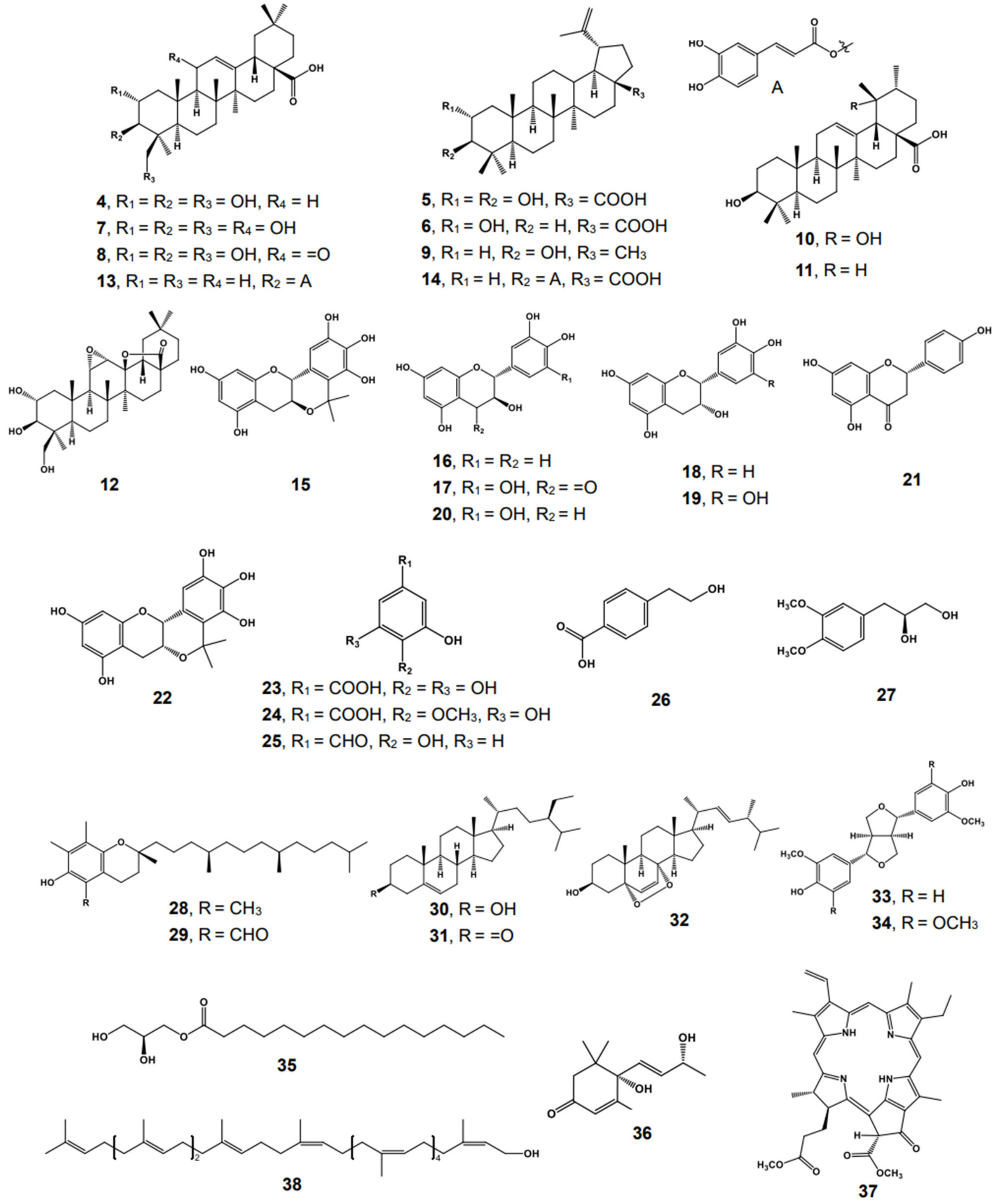

Figure S1. Structures of known compounds 4–38.

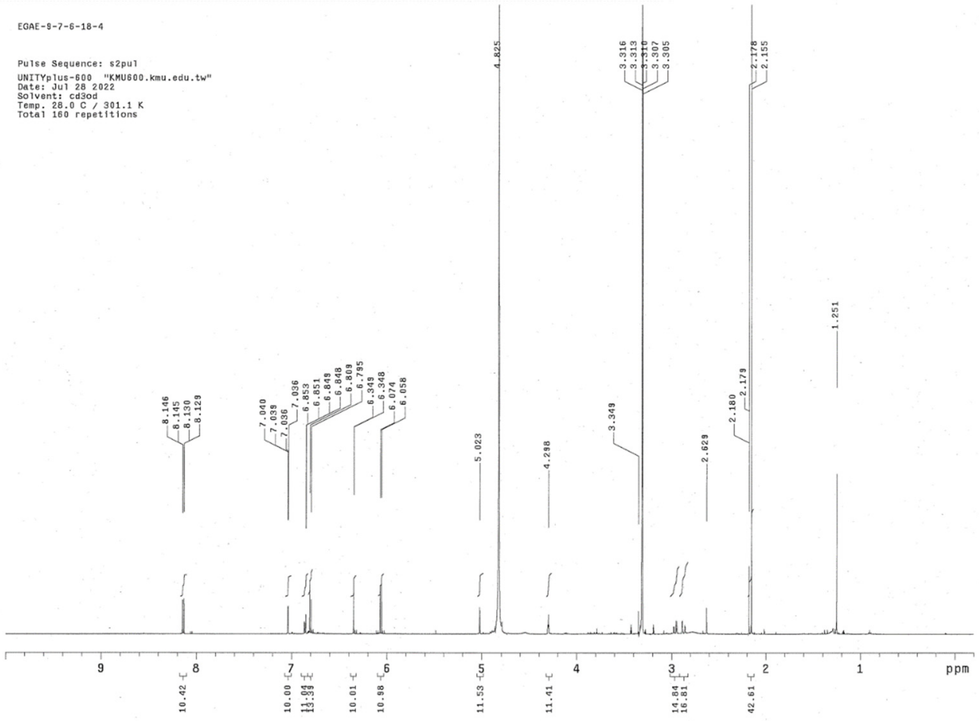

Figure S2. ^{1}**H NMR (600 MHz,** CD_3OD**) spectrum of 1.**

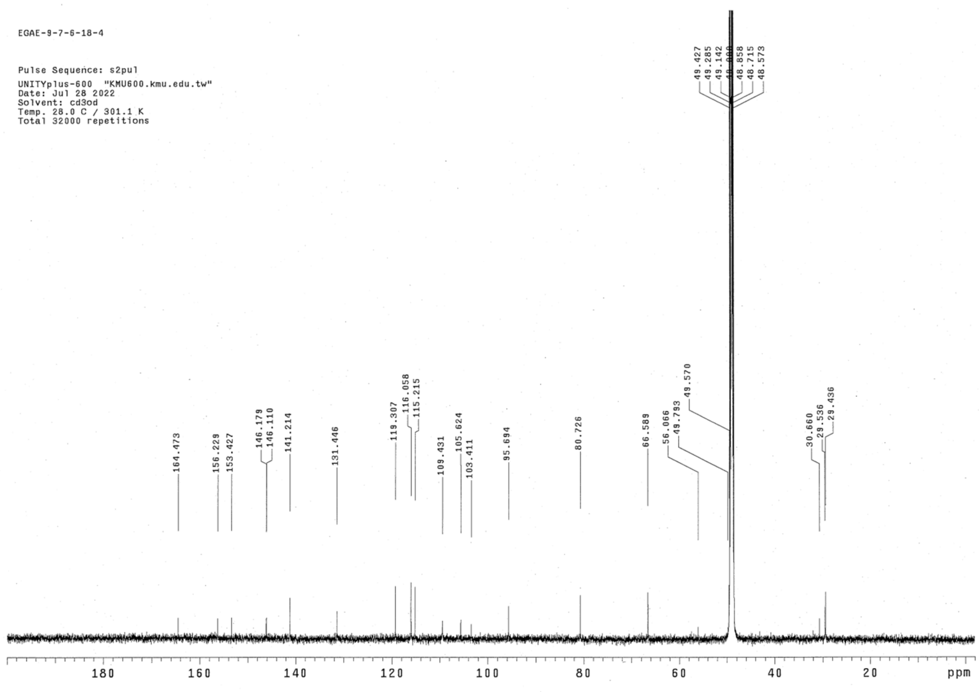

Figure S3. ^{13}**C NMR (150 MHz,** CD_3OD**) spectrum of 1.**

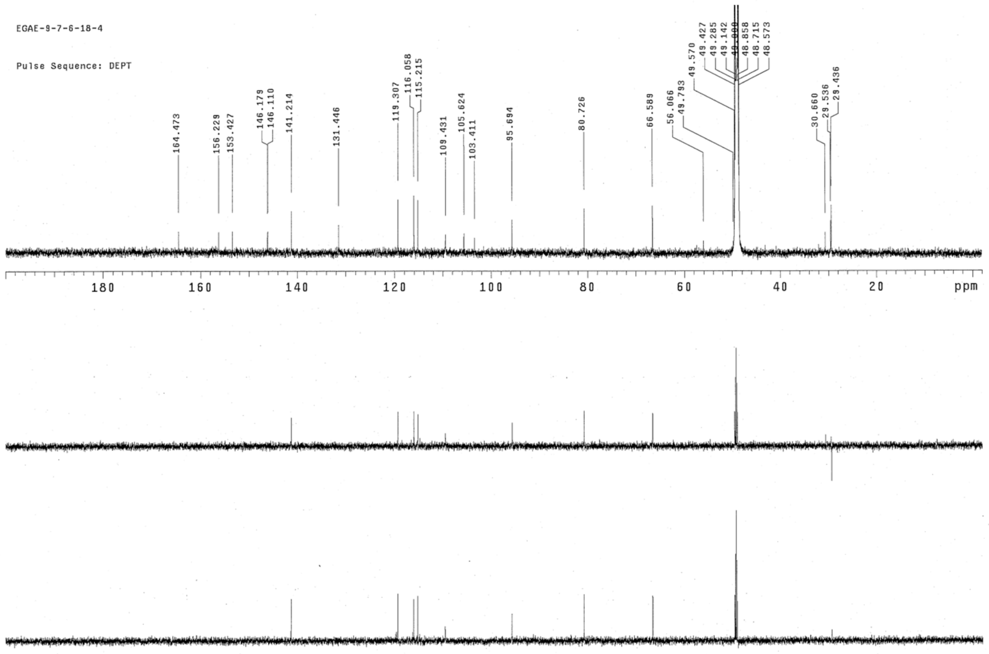

Figure S4. DEPT spectrum of 1.

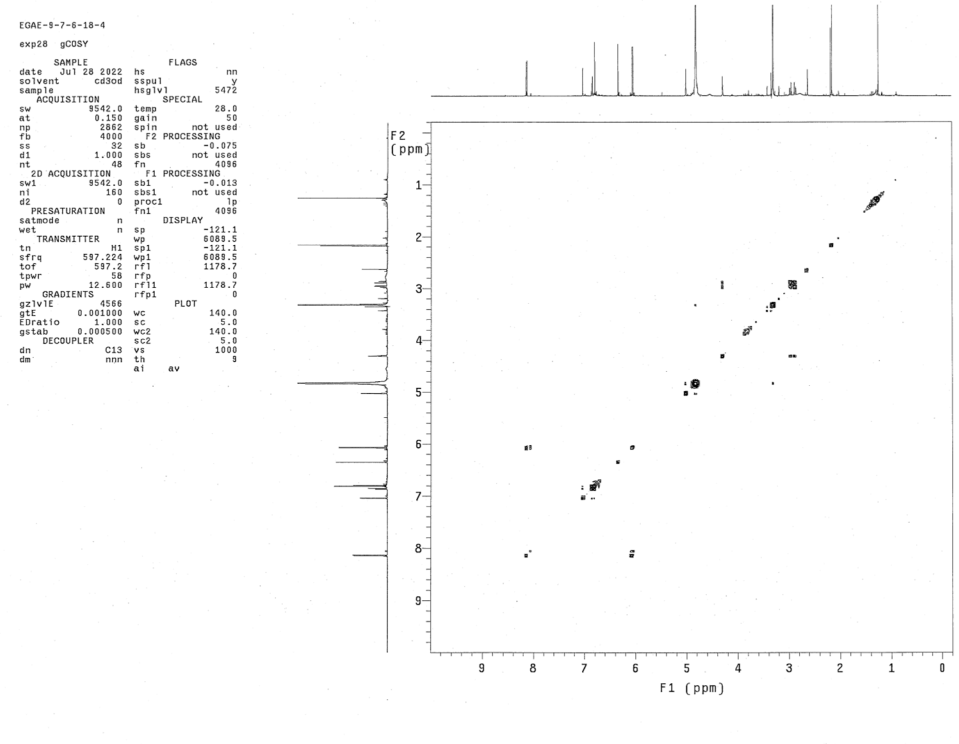

Figure S5. COSY spectrum of 1.

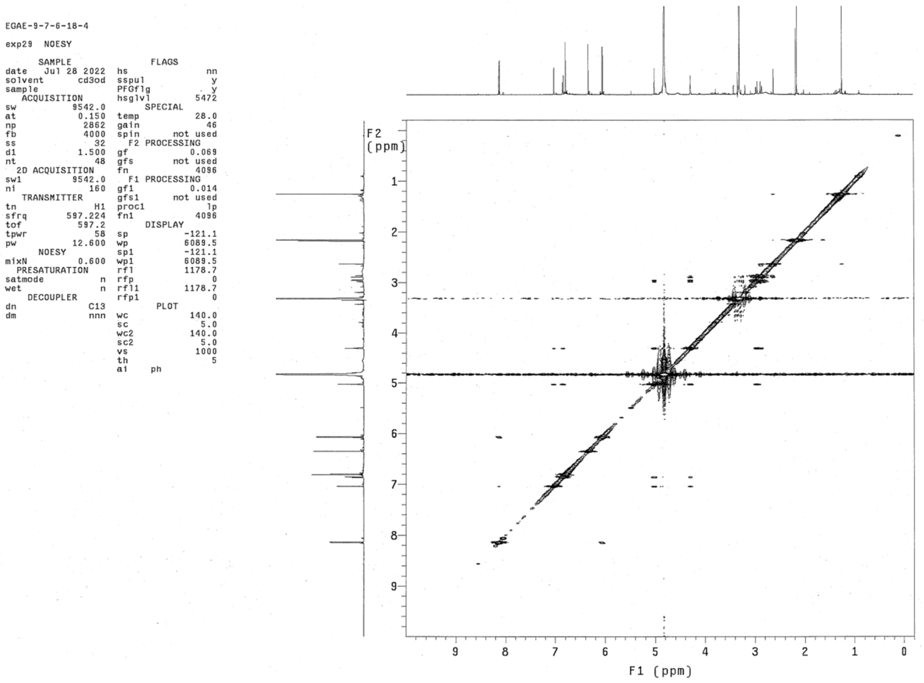

Figure S6. NOESY spectrum of 1.

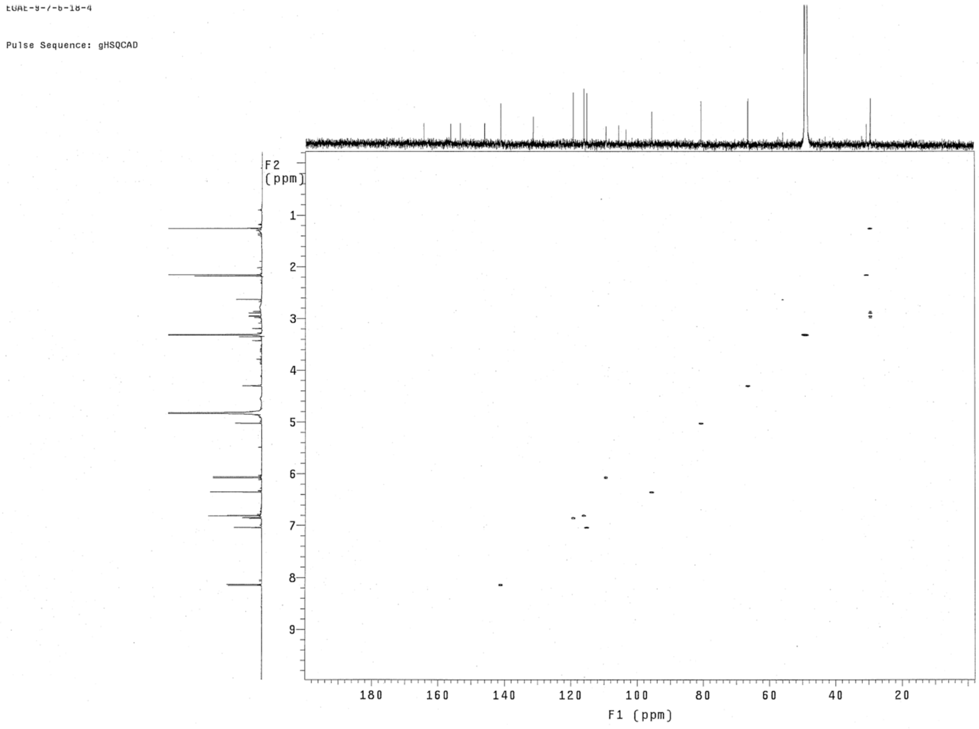

Figure S7. HSQC spectrum of 1.

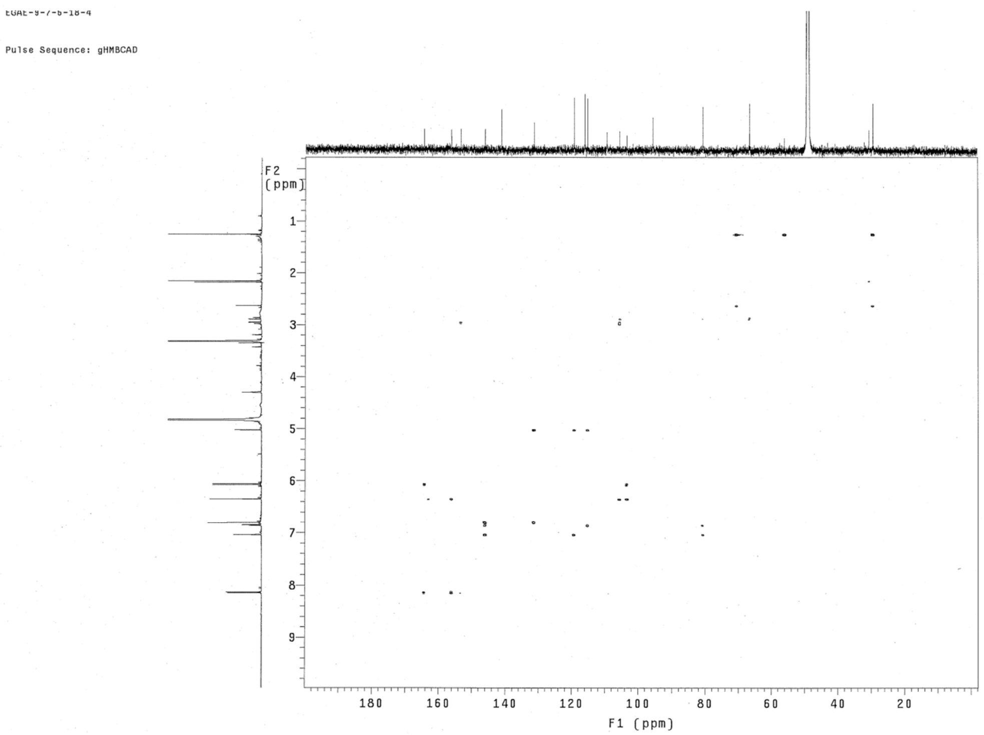

Figure S8. HMBC spectrum of 1.

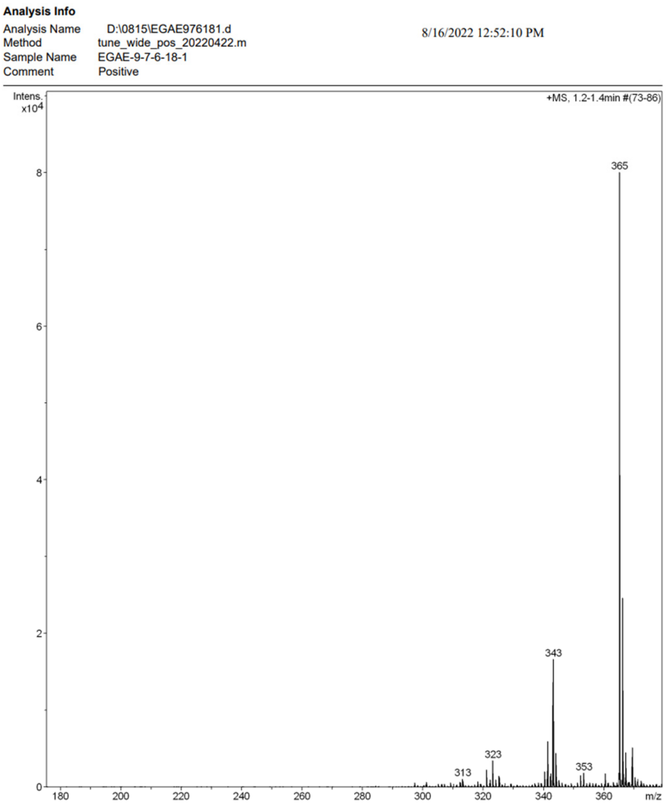

Figure S9. ESIMS spectrum of 1.

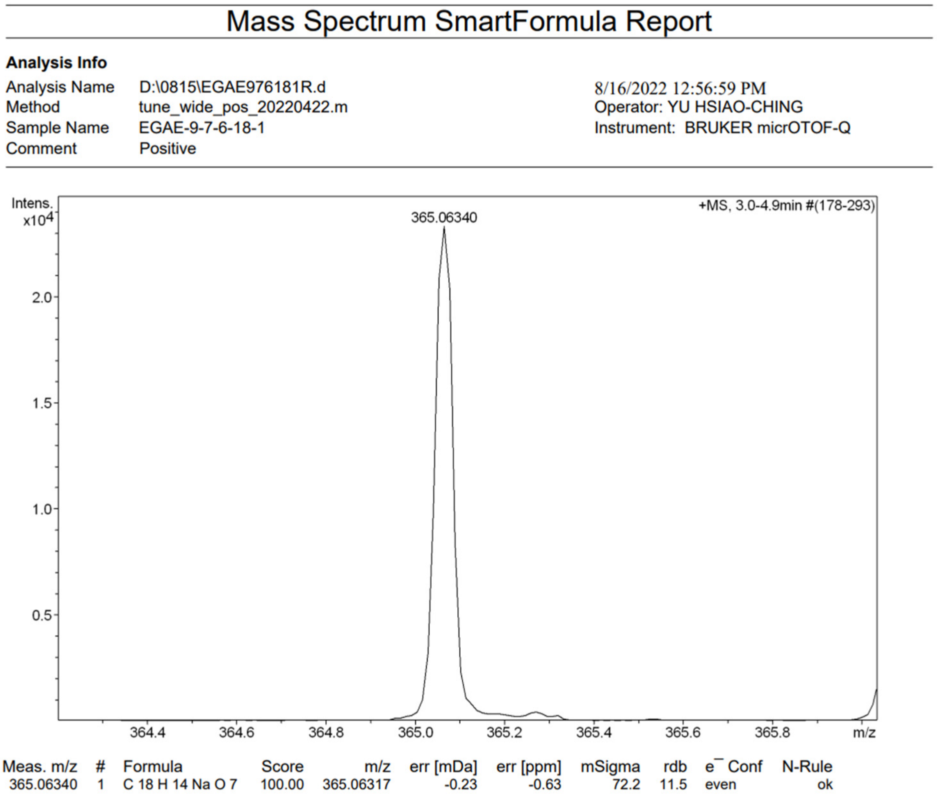

Figure S10. HRESIMS spectrum of 1.

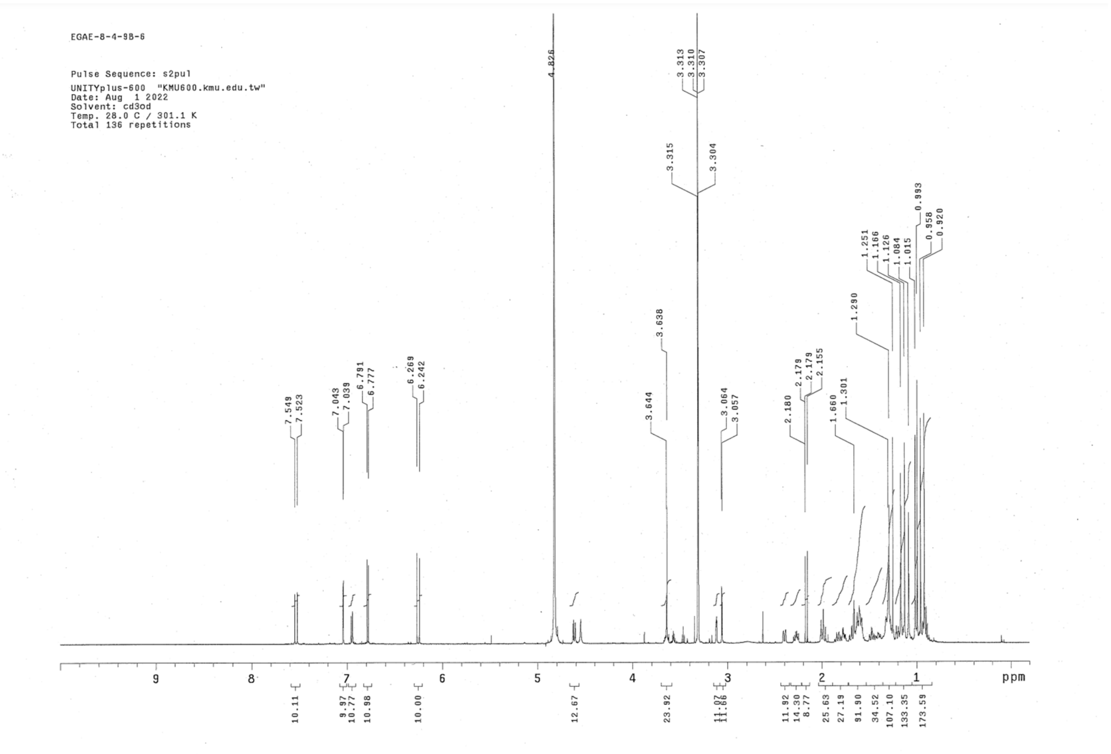

Figure S11. ^{1}H NMR (600 MHz, CD_3OD) spectrum of 2.

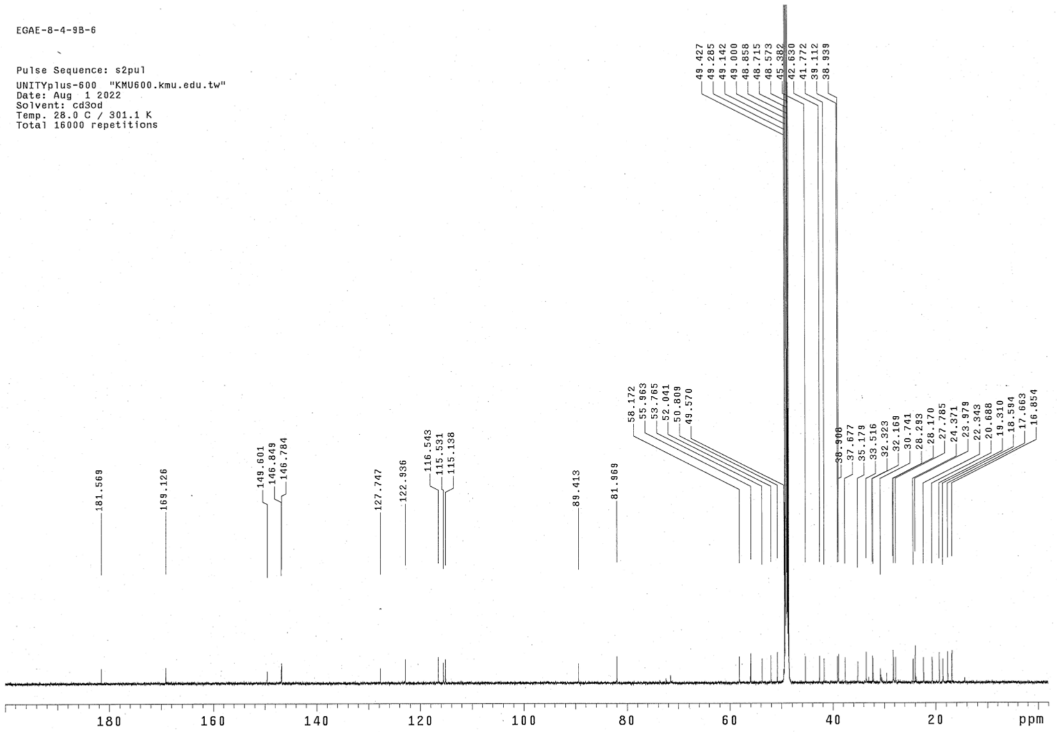

Figure S12. ^{13}C NMR (150 MHz, CD_3OD) spectrum of 2.

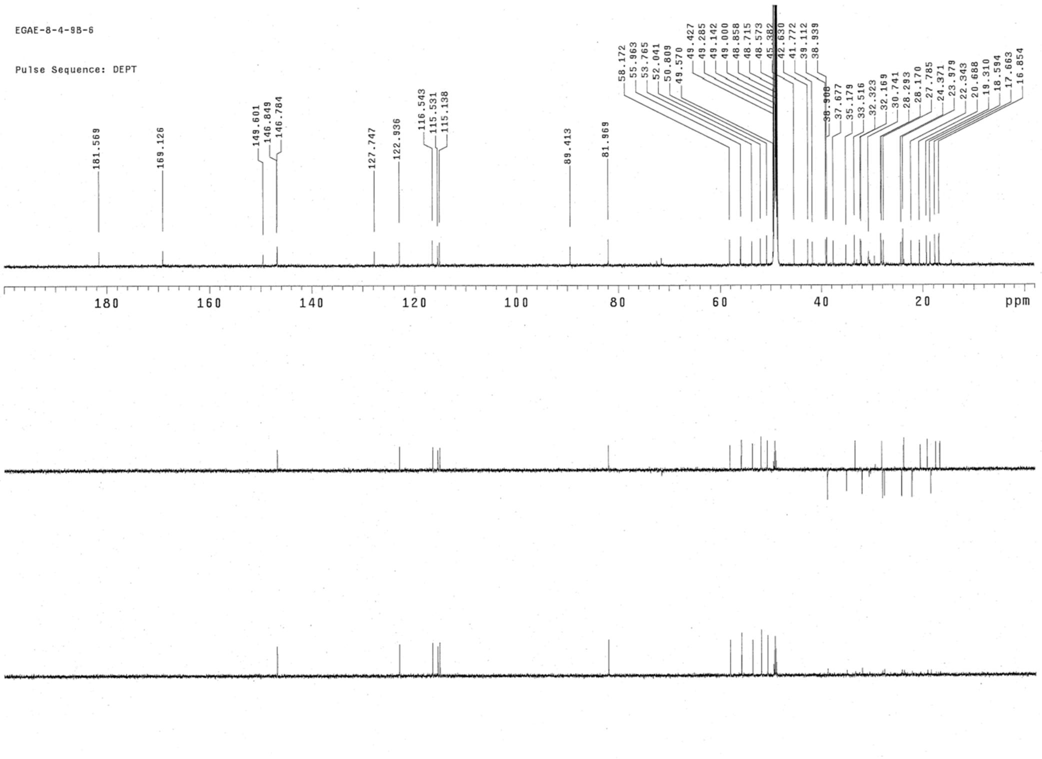

Figure S13. DEPT spectrum of 2.

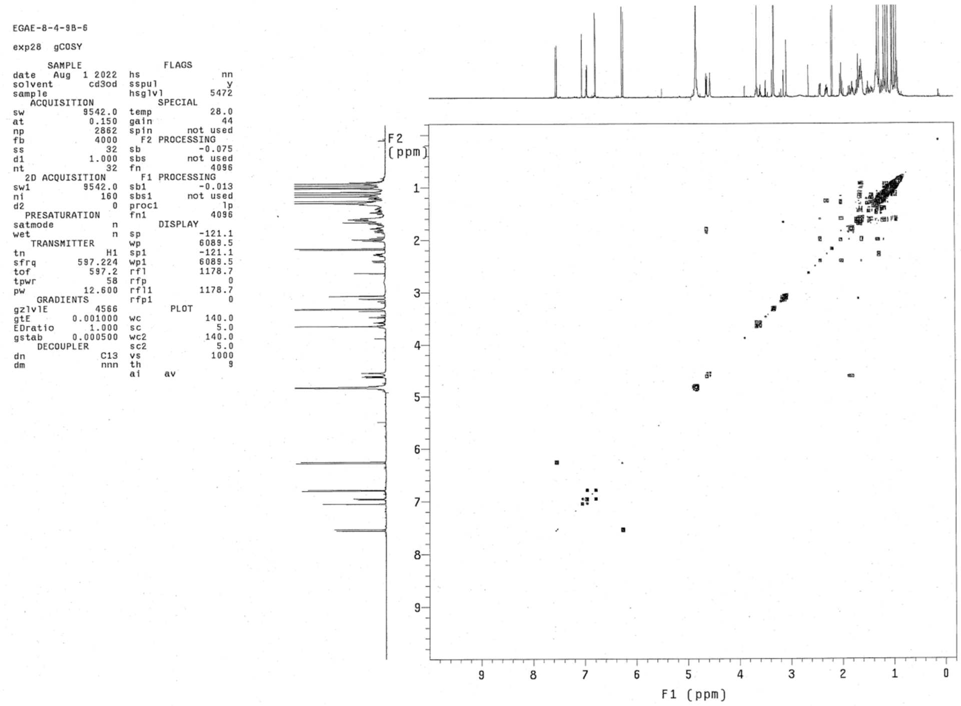

Figure S14. COSY spectrum of 2.

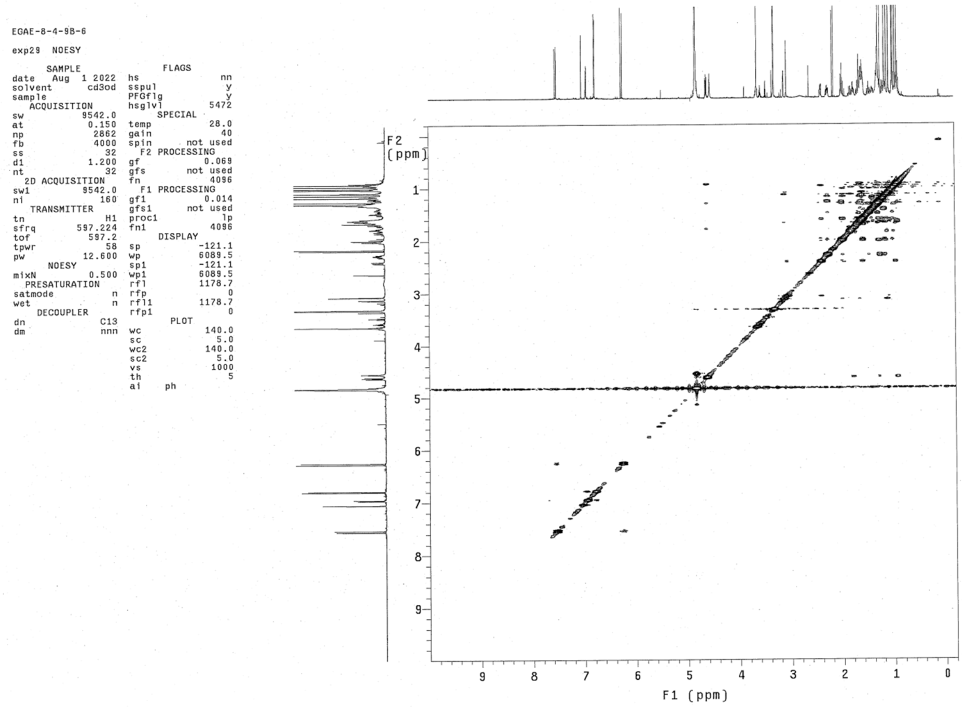

Figure S15. NOESY spectrum of 2.

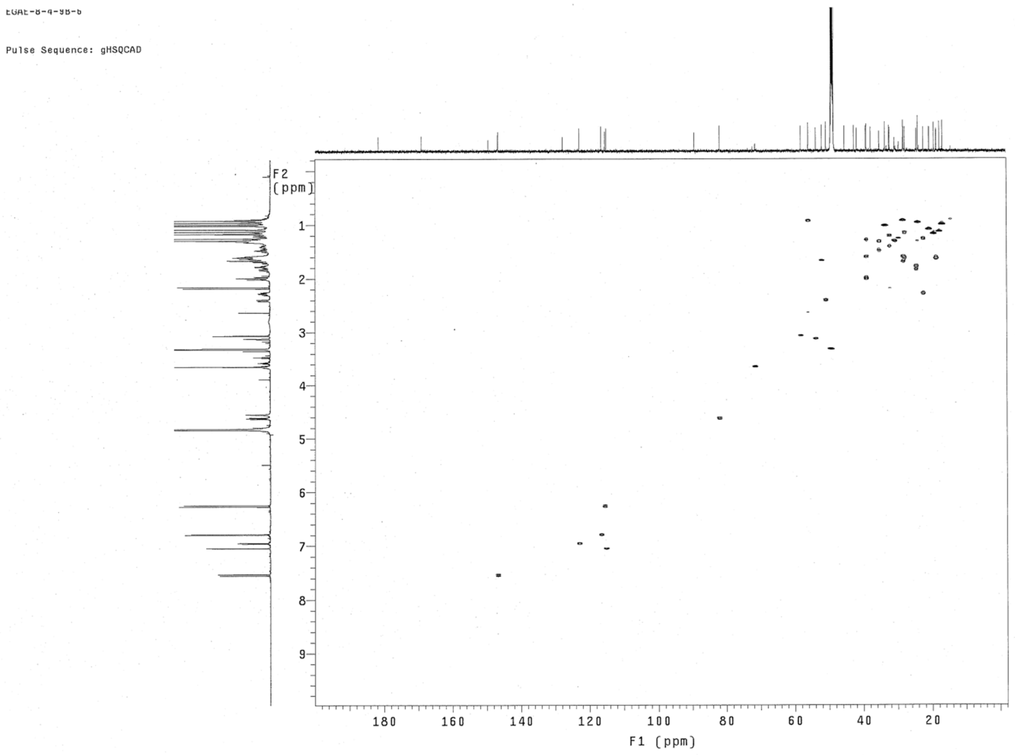

Figure S16. HSQC spectrum of 2.

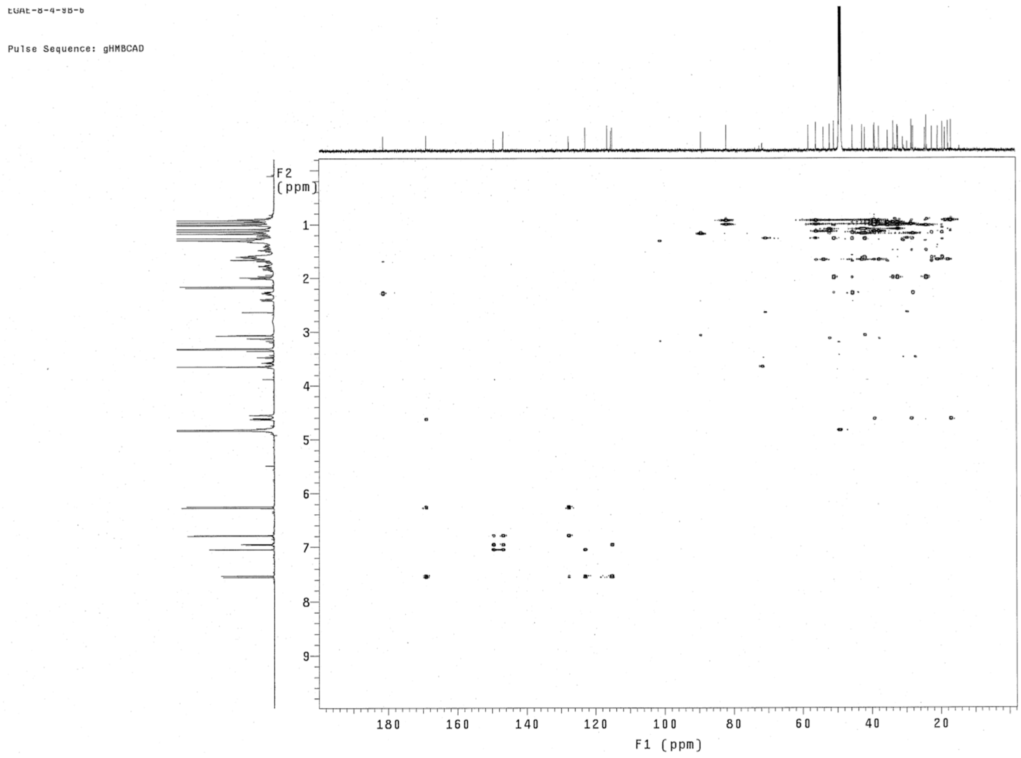

Figure S17. HMBC spectrum of 2.

Analysis Info

Analysis Name	D:\0816\EGAE849B6R1.d	8/17/2022 12:28:23 PM
Method	tune_wide_pos_20220422.m	
Sample Name	EGAE 8-4-9-B-6	
Comment	Positive	

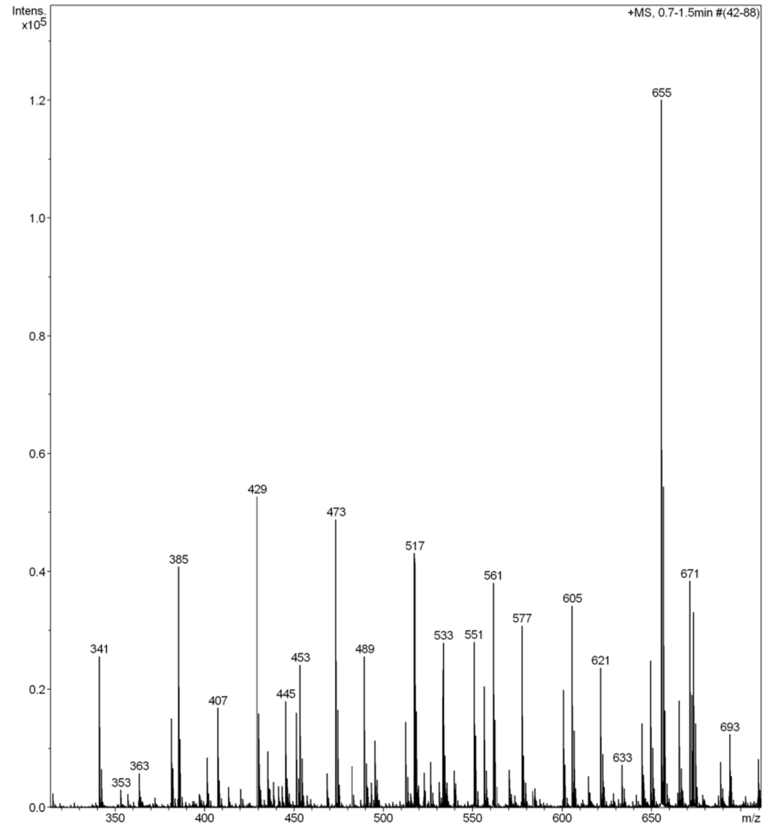

Figure S18. ESI spectrum of 2.

Mass Spectrum SmartFormula Report

Analysis Info

Analysis Name	D:\0816\EGAE849B6R3.d	8/17/2022 12:33:31 PM
Method	tune_wide_pos_20220422.m	Operator: YU HSIAO-CHING
Sample Name	EGAE 8-4-9-B-6	Instrument: BRUKER micrOTOF-Q
Comment	Positive	

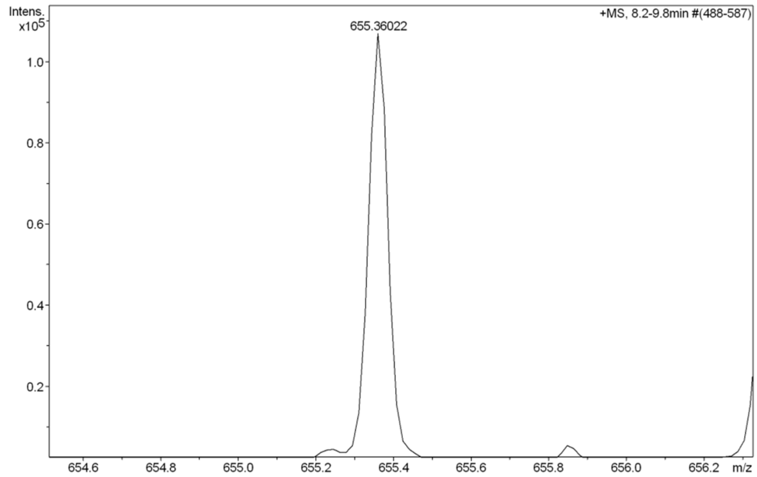

Meas. m/z	#	Formula	Score	m/z	err [mDa]	err [ppm]	mSigma	rdb	e⁻ Conf	N-Rule
655.36022	1	C 39 H 52 Na O 7	100.00	655.36053	0.31	0.47	25.8	13.5	even	ok

Figure S19. HRESI spectrum of 2.

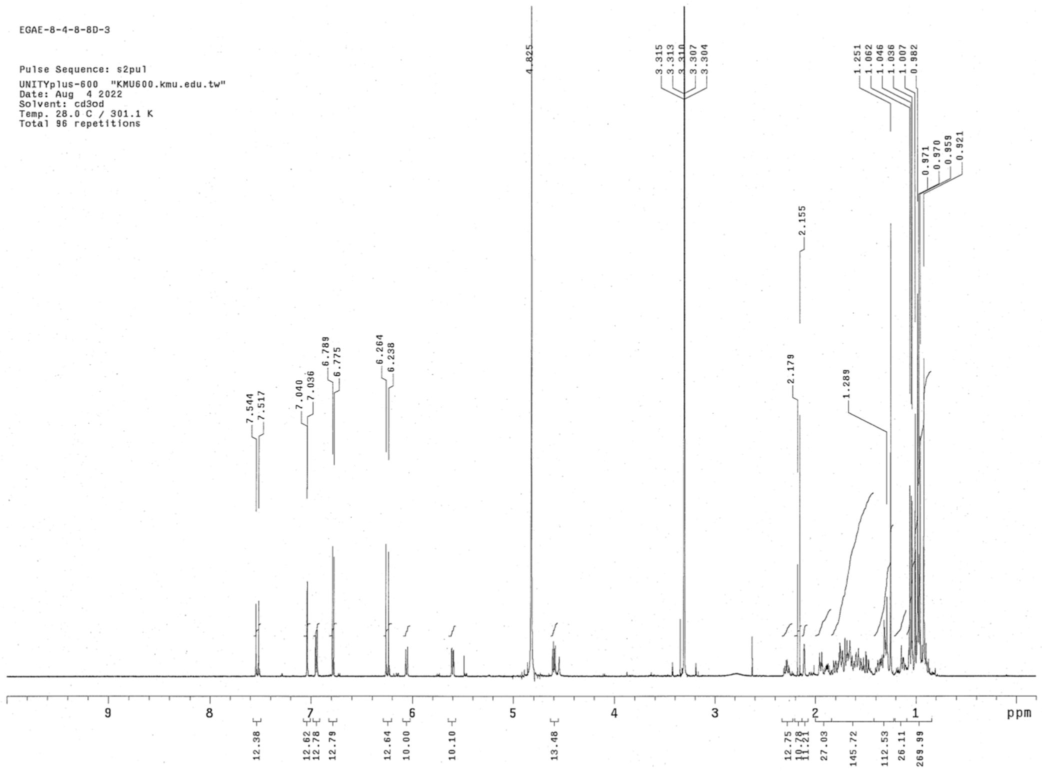

Figure S20. ^{1}H NMR (600 MHz, CD_3OD) spectrum of 3.

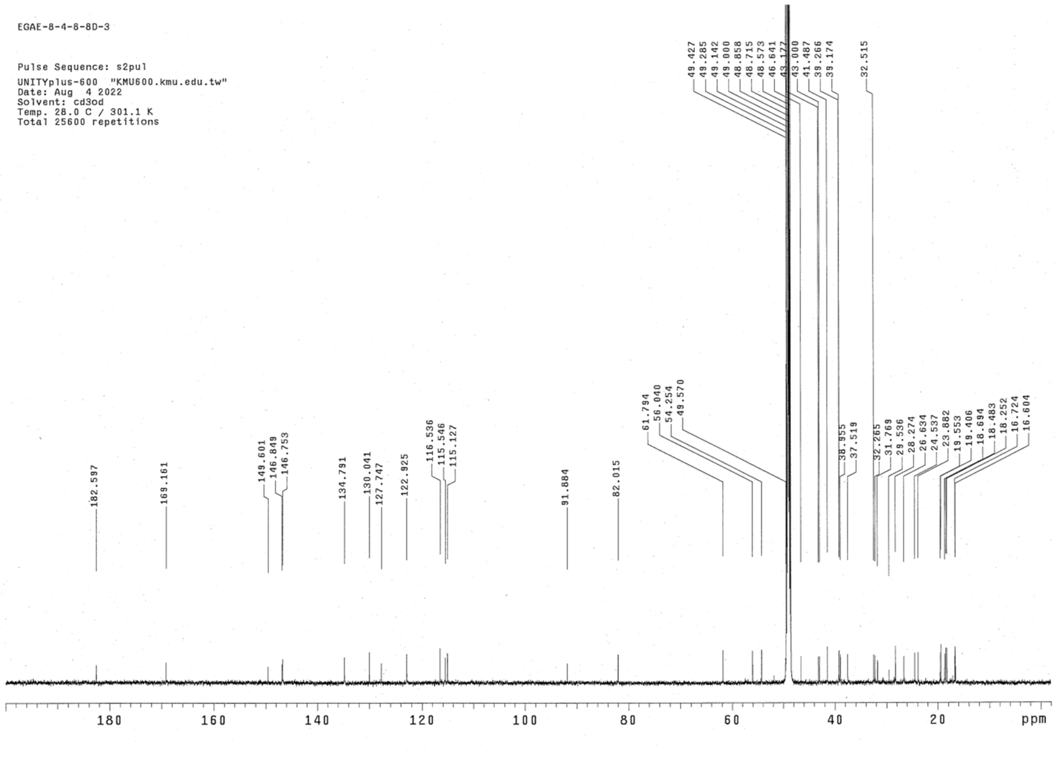

Figure S21. ^{13}C NMR (150 MHz, CD_3OD) spectrum of 3.

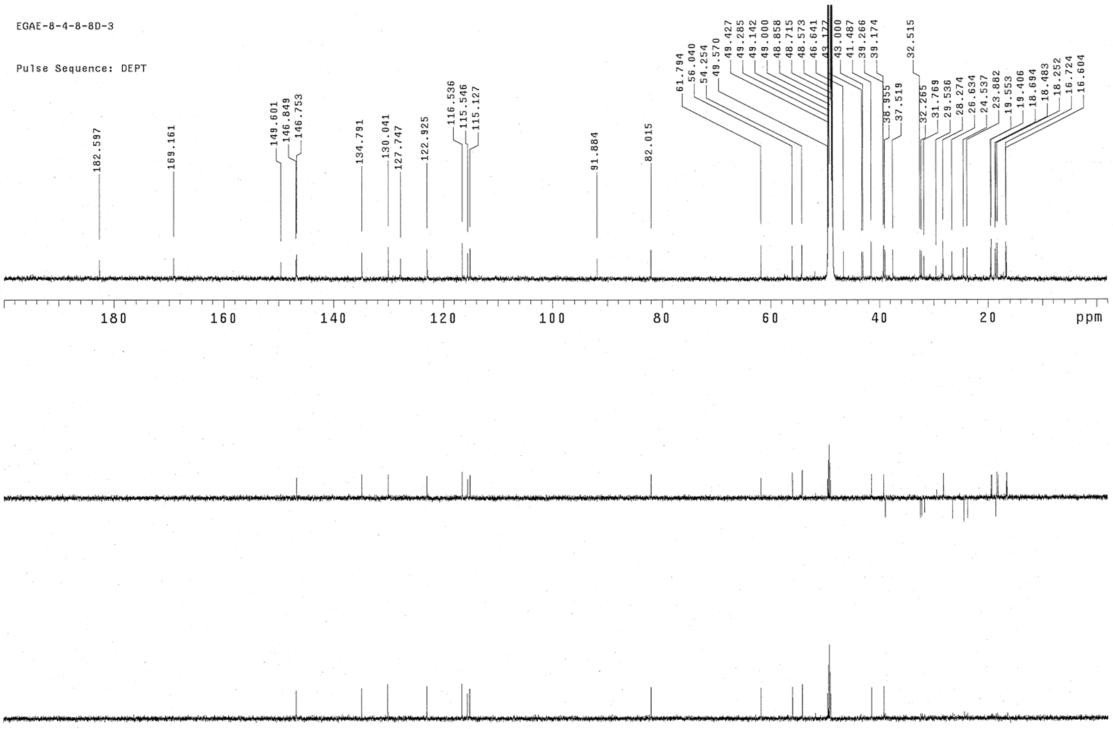

Figure S22. DEPT spectrum of 3.

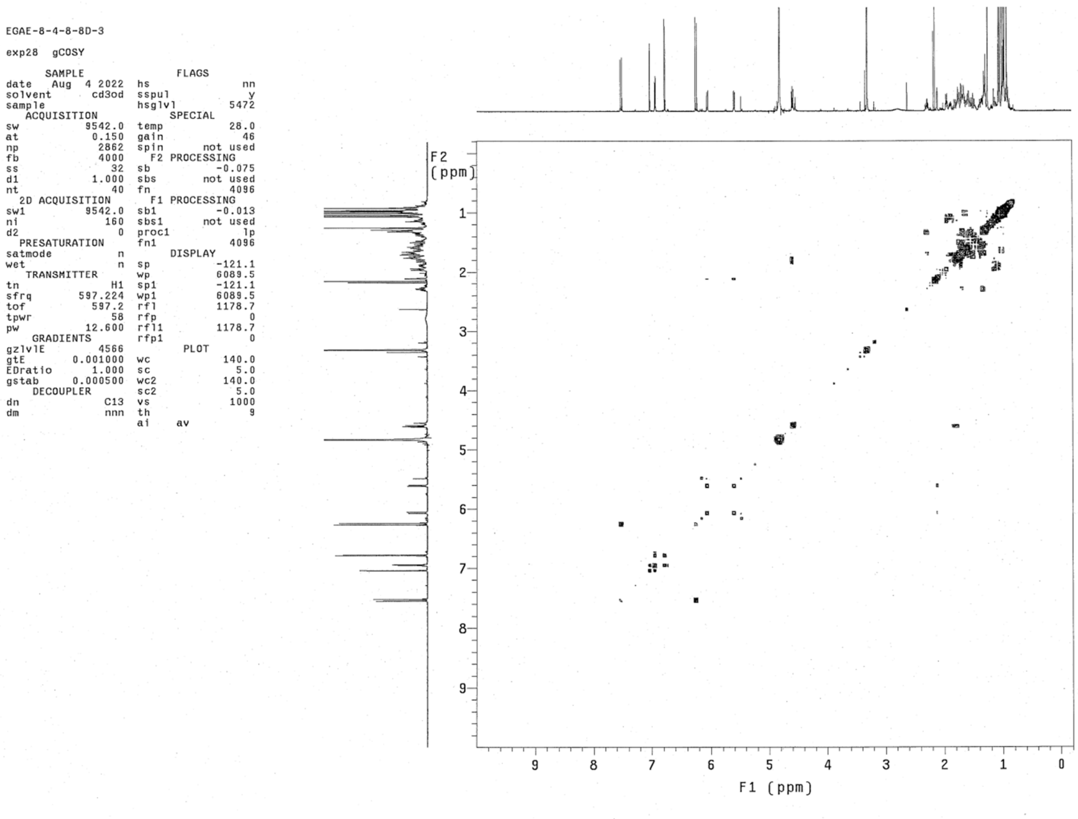

Figure S23. COSY spectrum of 3.

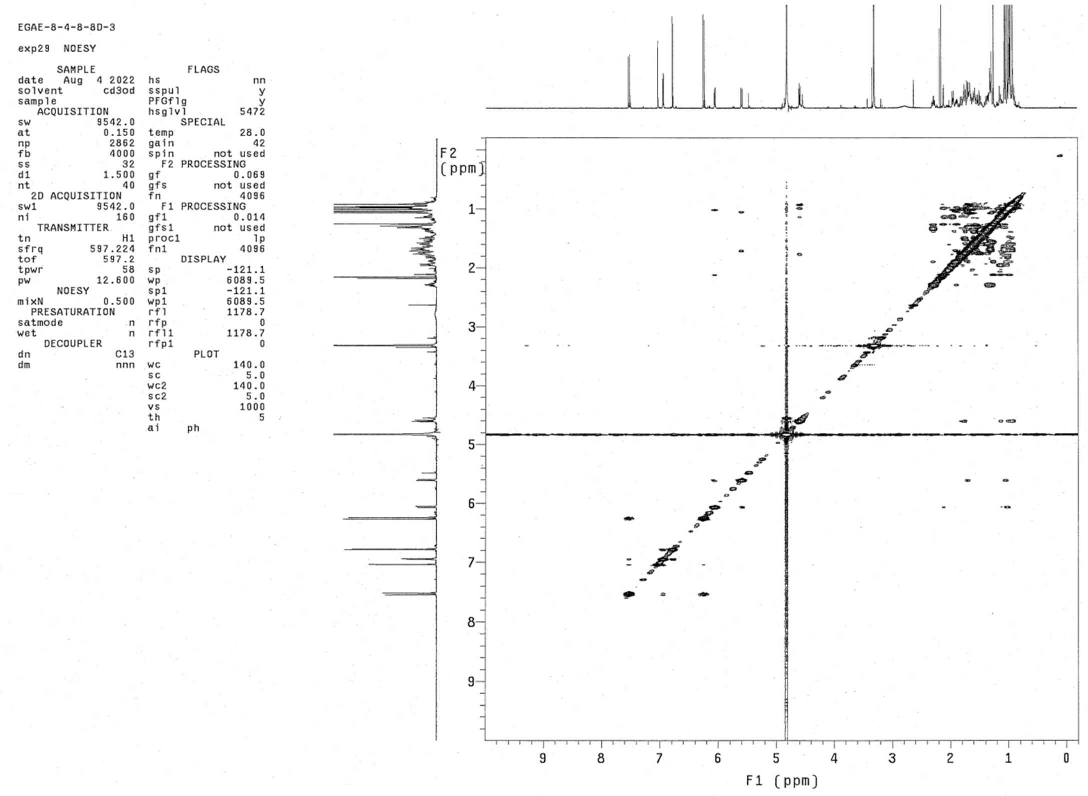

Figure S24. NOESY spectrum of 3.

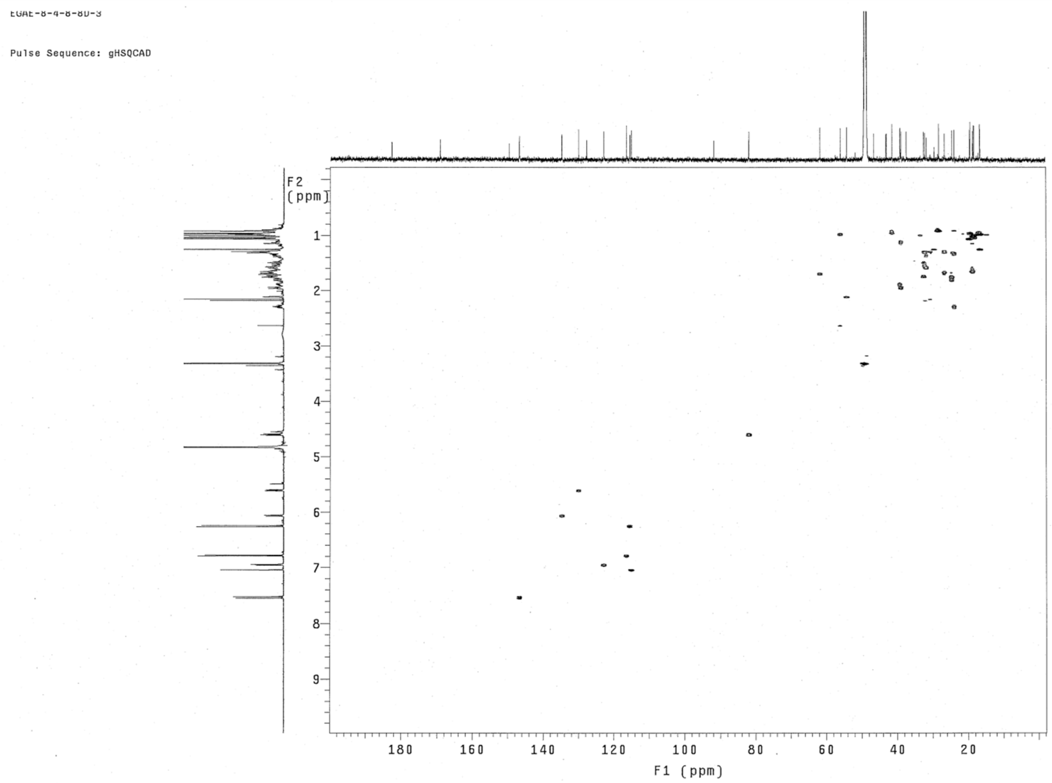

Figure S25. HSQC spectrum of 3.

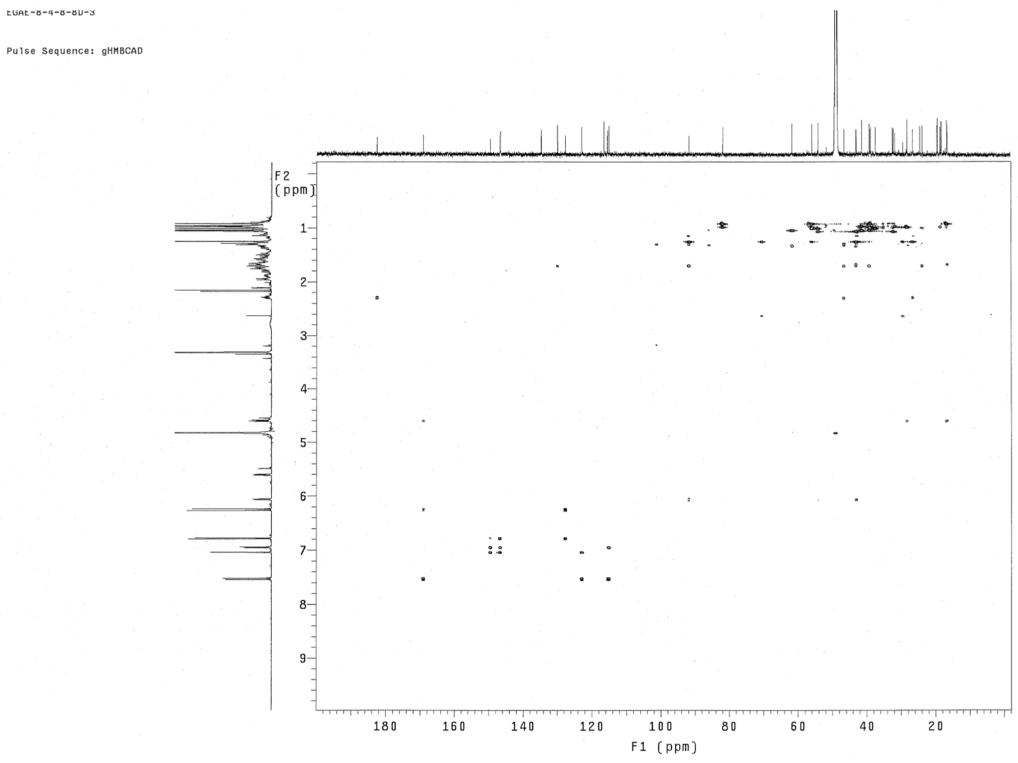

Figure S26. HMBC spectrum of 3.

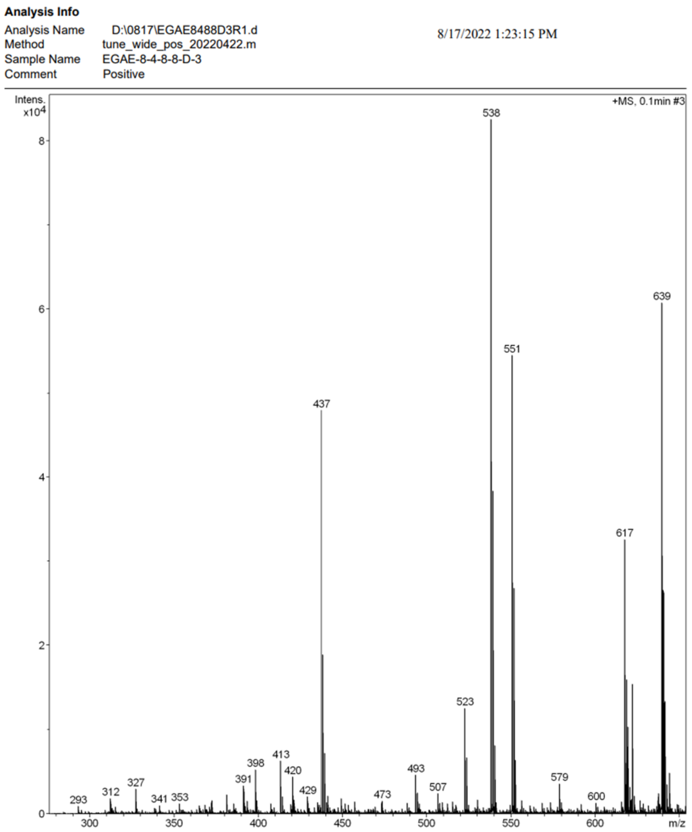

Figure S27. ESIMS spectrum of 3.

Mass Spectrum SmartFormula Report

Analysis Info

Analysis Name	D:\0817\EGAE8488D3R3.d	8/17/2022 1:26:23 PM	
Method	tune_wide_pos_20220422.m	Operator: YU HSIAO-CHING	
Sample Name	EGAE-8-4-8-8-D-3	Instrument: BRUKER micrOTOF-Q	
Comment	Positive		

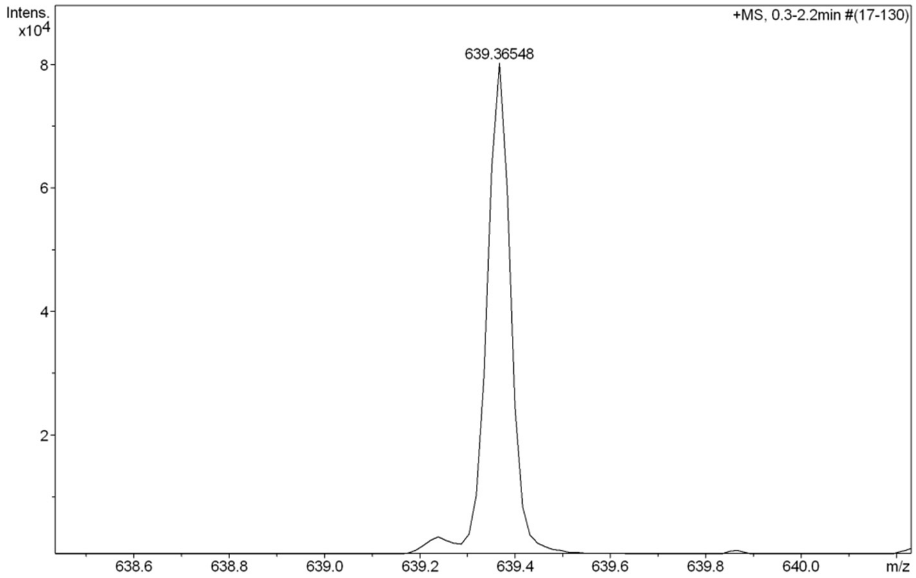

Meas. m/z	#	Formula	Score	m/z	err [mDa]	err [ppm]	mSigma	rdb	e⁻ Conf	N-Rule
639.36548	1	C 39 H 52 Na O 6	100.00	639.36561	0.13	0.20	61.5	13.5	even	ok

Figure S28. HRESIMS spectrum of 3.
